# Analysis of multiple databases identifies crucial genes correlated with prognosis of hepatocellular carcinoma

**DOI:** 10.1038/s41598-022-13159-4

**Published:** 2022-05-30

**Authors:** Zhifeng Lin, Xuqiong Huang, Xiaohui Ji, Nana Tian, Yu Gan, Li Ke

**Affiliations:** 1 Guangdong Province Key Laboratory of Major Obstetric Diseases, Department of Medical Record, The Third Affiliated Hospital of Guangzhou Medical University, Guangzhou, 510150 China; 2 Medical Administration Division, Affiliated Huadu Hospital, Southern Medical University (People’s Hospiatl of Huadu District), Guangzhou, 510800 China; 3 Department of Obstetrics and Gynaecology, Sun Yat-Sen Memorial Hospital, Sun Yat-Sen University, Guangzhou, 510120 China; 4 Department of Medical Record, The Fifth Affiliated Hospital of Guangzhou Medical University, Guangzhou, 510150 China

**Keywords:** Cancer, Genetics, Immunology

## Abstract

Despite advancements made in the therapeutic strategies on hepatocellular carcinoma (HCC), the survival rate of HCC patient is not satisfactory enough. Therefore, there is an urgent need for the valuable prognostic biomarkers in HCC therapy. In this study, we aimed to screen hub genes correlated with prognosis of HCC via multiple databases. 117 HCC-related genes were obtained from the intersection of the four databases. We subsequently identify 10 hub genes (JUN, IL10, CD34, MTOR, PTGS2, PTPRC, SELE, CSF1, APOB, MUC1) from PPI network by Cytoscape software analysis. Significant differential expression of hub genes between HCC tissues and adjacent tissues were observed in UALCAN, HCCDB and HPA databases. These hub genes were significantly associated with immune cell infiltrations and immune checkpoints. The hub genes were correlated with clinical parameters and survival probability of HCC patients. 147 potential targeted therapeutic drugs for HCC were identified through the DGIdb database. These hub genes could be used as novel prognostic biomarkers for HCC therapy.

## Introduction

Hepatocellular carcinoma (HCC), the second major cause of cancer-associated death worldwide, is a common cancer with poor prognosis due to its high mortality rate and complicated etiology^1,2^. To some extent, despite advancements made in the therapeutic strategies on HCC, such as surgical resection, transarterial chemoembolization, transplantation and radiofrequency ablation, the survival rate of HCC patient is not satisfactory enough^3–6^. Effective treatment interventions for HCC are urgently needed to improve their survival rate and quality of life^7,8^. Therefore, the identification of novel therapeutic targets and biomarkers will play a vital role in HCC treatment.

Currently, alpha-fetoprotein and des-gamma-carboxyprothrombin have been widely used as prognostic biomarkers in detecting HCC. However, its sensitivity is far from satisfactory^9,10^. Therefore, it is urgent to identify novel biomarkers for HCC therapy. Several studies have demonstrated that bioinformatics analysis can be used to identify valuable functional genes that could work as prognostic biomarkers^11–13^. Especially, identification of immune-related genes may contribute to HCC therapy. There have been increased immune-related genes in anti-tumour immune responses. For example, several investigators have found that inhibitors of cytotoxic T lymphocyte-associated antigen 4, programmed death-ligand 1 and programed death-1 induce anti-tumoral effects against HCC cells^14–16^. However, only a minority of patients benefits from immunotherapy, emphasizing the need to identify more effective hub genes associated with HCC.

In the current study, we screened out common genes through the intersection of 4 public databases. Then, we performed function enrichment analyses and protein–protein interaction (PPI) network of these genes. We subsequently identified the top ten hub genes by Cytoscape software. Next, we evaluated the correlation between hub genes and prognosis of HCC patients. The potential associations between the hub genes and immune infiltration cells in HCC were also explored. Finally, we obtain potential targeted therapeutic drugs for HCC through DGIdb.

## Materials and methods

### Ethics statement

Because the current study strictly followed the online database publication guidelines and data access policies, approval from an ethics committee was not required. All methods were performed in accordance with the relevant guidelines and regulations.

### Data source

All the data analyzed in this study were derived from public databases. GeneCards is an integrative database that provides information on human genes^17^. DISEASES, a weekly updated web database, integrates information on gene-disease associations from manually curated literature, cancer mutation data, automatic text mining and genome-wide association studies from existing databases^18^. Comparative Toxicogenomics Database (CTD), a publicly available database, aims to provide environmental exposure information on gene–disease, chemical–disease and chemical–gene/protein interactions relationships that affects human health^19^. Online Mendelian Inheritance in Man (OMIM), a knowledgebase of human genes and phenotypes, is frequently updated and freely accessible^20^. HCC-related genes were extracted from GeneCards (https://www.genecards.org/), DISEASES (https://diseases.jensenlab.org/Search), CTD (http://ctdbase.org/) and OMIM (https://www.omim.org/) with the keyword “hepatocellular carcinoma”.

### Common gene

The common genes for HCC were obtained by the intersection of the four databases in the Venn diagram online construction website (https://bioinfogp.cnb.csic.es/tools/venny/index.html). All of these common genes were included for further analysis.

### Enrichment analysis

Gene Ontology (GO) and Kyoto Encyclopedia of Genes and Genomes (KEGG) pathway enrichment analyses were conducted for the identified common genes by using Metascape (https://metascape.org/)^21^.

### PPI network construction and hub gene identification

The Search Tool for the Retrieval of Interacting Genes (STRING) database (http://string-db.org/) was selected to construct the protein–protein interaction (PPI) network^22^. The common genes identified previously were uploaded to the STRING database to evaluate the potential PPI relationship. PPI pairs with a combined score more than 0.4 were extracted. Subsequently, the PPI network construction was visualized by Cytoscape software^23^. Nodes with a high degree tended to be act as an important role in the network. CytoHubba was used to calculate the degree of each node. The top ten genes were then identified as hub genes by the rank of degree.

### UALCAN analysis

UALCAN (http://ualcan.path.uab.edu) provides relative transcriptional expression of potential genes between normal and tumor samples as well as association of relative clinical parameters with the transcriptional expression^24^. In our study, UALCAN was used to perform the mRNA expression of the hub genes in primary HCC tissues and normal control tissues. The relationships between hub genes and clinical parameters were also explored. *P* < 0.05 (Students t-test) was considered significant.

### HCCDB analysis

HCCDB (http://lifeome.net/database/hccdb/home.html), a publicly available web-based database, owns 15 public HCC gene expression datasets to offer a one-stop resource for gene expression analysis in HCC^25^. We used HCCDB database to validate whether hub genes expression achieved statistical significance in HCC. *P* < 0.05 was considered significant.

### Human protein atlas

The Human Protein Atlas (https://www.proteinatlas.org) is based on immunohistochemistry data of proteins expression^26,27^. In this study, we obtained immunohistochemistry image for four hub genes from this database.

### Kaplan–Meier plotter analysis

The Kaplan–Meier plotter (http://kmplot.com/analysis/) is able to evaluate the effect of hub genes on survival probability in HCC patients. Log rank P-value and hazard ratio (HR, and 95% confidence intervals) were computed^28^. In this study, the associations between the expression of hub genes and survival state (including overall survival, OS; progression free survival, PFS; recurrence-free survival, RFS; disease free survival, DSS) were analyzed by Kaplan–Meier plotter. We also explored the prognostic value of hub genes in HCC who received sorafenib treatment. Additionally, we demonstrated the prognostic value of combinatory mRNA expression of all ten hub genes in HCC patients and clinical parameters. P < 0.05 was considered significant.

### cBioPortal analysis

Multidimensional cancer genomics data sets are available from cBioPortal (http://cbioportal.org)^29^, we performed all hub genes alterations in the LIHC sample (MSK, Clin Cancer Res 2018; INSERM, Nat Genet 2015; MSK, PLOS One 2018; AMC, Hepatology 2014; RIKEN, Nat Genet 2012; TCGA, Firehose legacy). We explored the genetic alterations of hub genes in per sample by OncoPrint and the prognostic value of these hub genes in OS and DSS of HCC.

### TIMER2.0 analysis

TIMER2.0, a comprehensive resource of online server, provides a systematical analysis of immune infiltrates across various cancer types^30^. In this study, we conducted the associations of hub genes expression with HCC related immune cells, including CD8+ T cells, CD4+ T cells, macrophages, B cells, dendritic cells (DCs) and neutrophils. *P* < 0.05 was considered significant. Furthermore, we performed the role of hub genes expression combined with macrophage level on OS in HCC patient. The multivariate Cox proportional hazard models of ten hub genes were constructed by adjusted for tumor stage, age, race, gender, macrophage level and tumor purity.

### GEPIA

We used GEPIA (http://gepia.cancer-pku.cn/index.html) to analyze the associations between hub genes and immune checkpoints (CD274, CTLA4 and PDCD1)^31^. Spearman correlation coefficient was used to assess the relationships between hub genes and immune checkpoints expressions in HCC. P < 0.05 was the threshold for significance.

### Drug screening

In order to obtain potential targeted therapeutic drugs for HCC, the hub genes were imported into the DGIdb (http://www.dgidb.org)^32,33^ to acquire potential HCC-associated treatment drugs with the preset filters selected “Approved”.

## Results

### Identification of common genes

**Figure 1:**
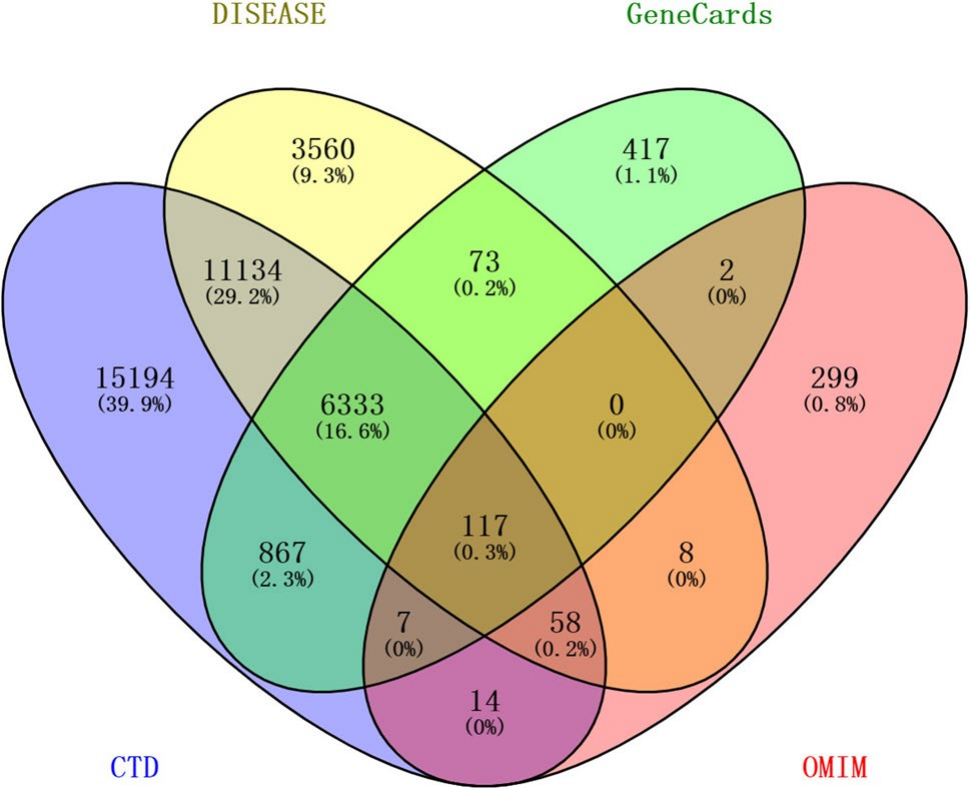
Venn Diagram of intersection between all the genes of HCC obtained from 4 public databases.

For the purpose of acquiring common genes, we obtained HCC-related genes available in GeneCards, DISEASES, CTD and OMIM databases. After removing the duplicate genes, there were 7816 HCC-related genes in GeneCards, 21283 HCC-related genes in DISEASES, 33724 HCC-related genes in CTD and 505 HCC-related genes in OMIM. Ultimately, 117 common genes were identified by the intersection of the four databases. The Venn Diagram of intersection between all HCC-related genes obtained from these 4 databases was showed in Fig. 1. These 117 genes were listed in Supplementary Table S1.

### GO and KEGG enrichment analyses of the common genes

**Figure 2:**
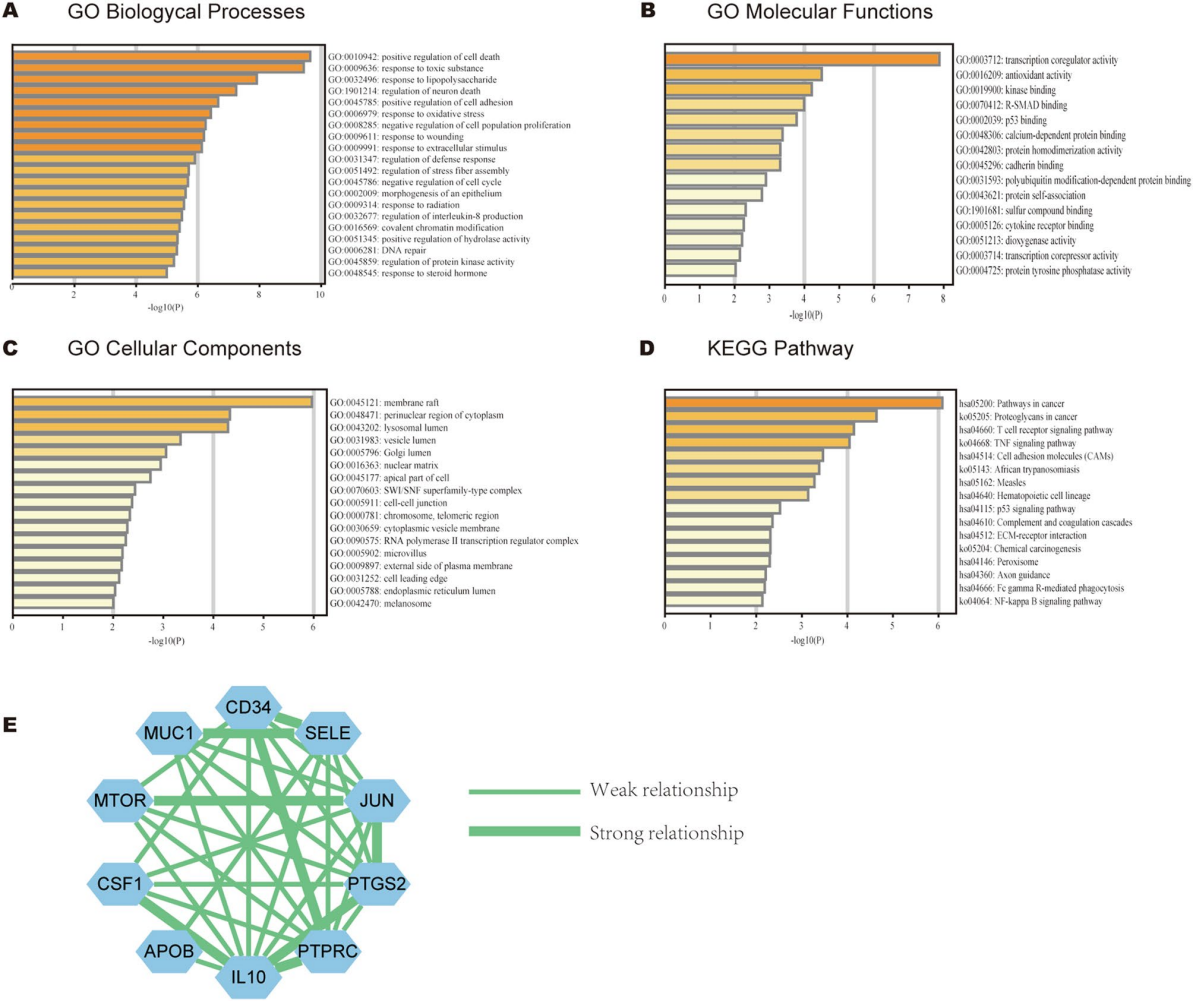
GO, KEGG and PPI network. (**A**–**C**) GO enrichment analysis with common genes, (**D**) KEGG pathway analysis with common genes, (**E**) PPI network of hub genes.

Biological processes (BP) analysis indicated that 117 common genes were dramatically enriched in positive regulation of cell death, response to toxic substance, response to lipopolysaccharide and positive regulation of cell adhesion (Fig. 2A). Molecular functions (MF) demonstrated that the common genes were significantly enriched in transcription coregulatory activity, antioxidant activity, kinase binding and R-SMAD binding (Fig. 2B). Cellular components (CC) showed that the common genes were significantly concentrated in membrane raft, perinuclear region of cytoplasm, lysosomal lumen and vesicle lumen (Fig. 2C). Additionally, KEGG analysis revealed that all common genes were mainly enriched in pathways in cancer, proteoglycans in cancer, T cell receptor signaling pathway and TNF signaling pathway (Fig. 2D).

### PPI network construction and hub gene identification

PPI network with 97 nodes and 210 edges were visualized in the Cytoscape. Next, we used the connectivity degree to identify the top ten genes from the PPI network (Fig. 2E). Strong relationship between two genes indicated that their combined score was more than 0.7. Table 1 revealed that jun proto-oncogene (*JUN*) was the most prominent gene with the highest connectivity degree = 23, followed by interleukin 10 (*IL10*; degree = 22), CD34 molecule (*CD34*; degree = 15), mechanistic target of rapamycin kinase(*MTOR*; degree = 14), prostaglandin-endoperoxide synthase 2 (*PTGS2*; degree = 13), protein tyrosine phosphatase receptor type c (*PTPRC*; degree = 13), selectin e (*SELE*; degree = 12), colony stimulating factor 1 (*CSF1*; degree = 11), apolipoprotein b (*APOB*; degree = 10), mucin 1 (*MUC1*; degree = 10).

**Table 1:** Top ten hub genes with higher degree of connectivity.

Gene symbol	Gene description	Degree
JUN	Jun proto-oncogene	23
IL10	Interleukin 10	22
CD34	CD34 molecule	15
MTOR	Mechanistic target of rapamycin kinase	14
PTGS2	Prostaglandin-endoperoxide synthase 2	13
PTPRC	Protein tyrosine phosphatase receptor type C	13
SELE	Selectin E	12
CSF1	Colony stimulating factor 1	11
APOB	Apolipoprotein B	10
MUC1	Mucin 1	10

### Hub gene expression in HCC

**Figure 3:**
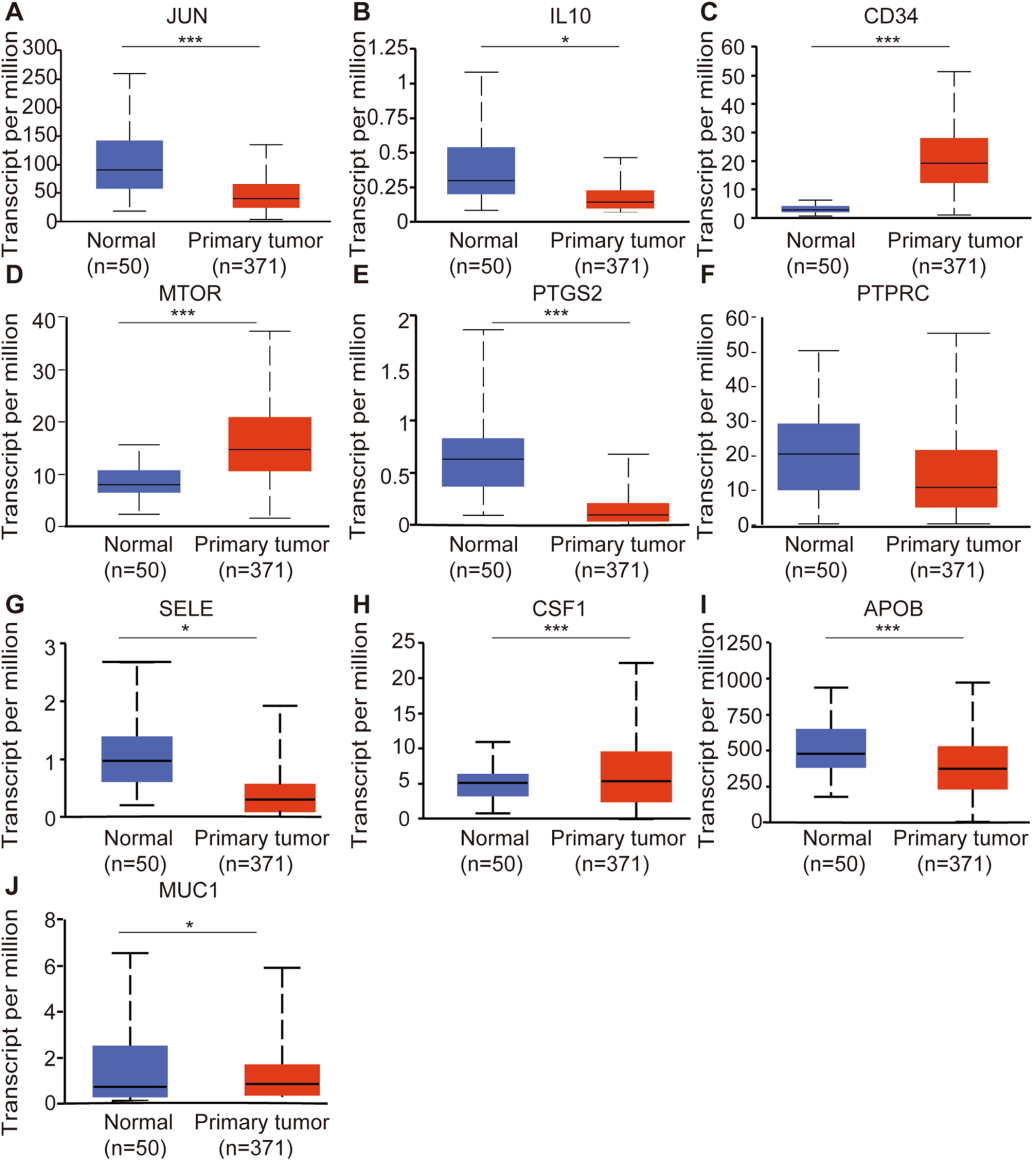
mRNA expression of hub genes in HCC tissues and adjacent normal liver tissues. (**A**) JUN, (**B**) IL10, (**C**) CD34, (**D**) MTOR, (**E**) PTGS2, (**F**) PTPRC, (**G**) SELE, (**H**) CSF1, (**I**) APOB, (**J**) MUC1. *P < 0.05, **P < 0.01, ***P < 0.001.

**Figure 4:**
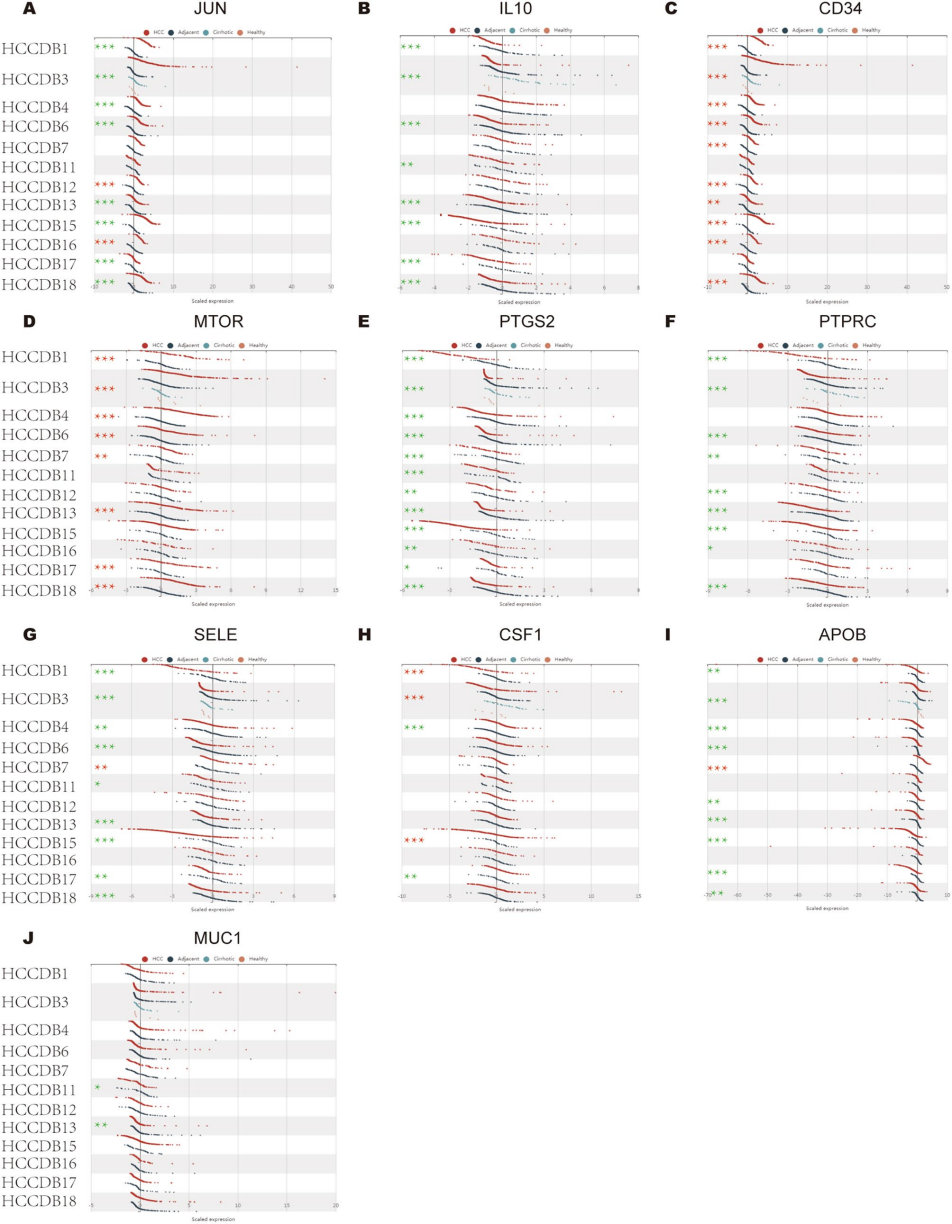
The relative expression of hub genes in normal and HCC samples. (**A**) JUN, (**B**) IL10, (**C**) CD34, (**D**) MTOR, (**E**) PTGS2, (**F**) PTPRC, (**G**) SELE, (**H**) CSF1, (**I**) APOB, (**J**) MUC1. *P < 0.05, **P < 0.01, ***P < 0.001. Note: Red asterisks represent upregulated genes in HCC patients, and green asterisks represent downregulated genes in HCC patients.

The mRNA expression of hub genes in HCC patients was subsequently explored by UALCAN. Among them, *JUN* (Fig. 3A), *IL10* (Fig. 3B), *PTGS2* (Fig. 3E), *SELE* (Fig. 3G), *APOB* (Fig. 3I) were significantly downregulated in HCC, while *CD34* (Fig. 3C), *MTOR* (Fig. 3D), *CSF1* (Fig. 3H) and *MUC1* (Fig. 3J) were upregulated. There was no significant difference in the expression of *PTPRC* (Fig. 3F) between HCC and normal tissues. Furthermore, the mRNA expression of hub genes in HCC, adjacent normal tissue, cirrhotic and healthy samples was acquired from HCCDB database (Fig. 4). *JUN* was confirmed to be downregulated in HCC tissues compared with adjacent normal tissues in HCCDB1, HCCDB3, HCCDB4, HCCDB6, HCCDB13, HCCDB15, HCCDB17, HCCDB18. The similar results showed for *IL10* (HCCDB1, HCCDB3, HCCDB6, HCCDB11, HCCDB13, HCCDB15, HCCDB17, HCCDB18), *PTGS2* (from HCCDB1 to HCCDB18), *SELE* (HCCDB1, HCCDB3, HCCDB4, HCCDB6, HCCDB11, HCCDB13, HCCDB15, HCCDB17, HCCDB18), *APOB* (HCCDB1, HCCDB3, HCCDB4, HCCDB6, HCCDB12, HCCDB13, HCCDB15, HCCDB17, HCCDB18), *PTPRC* (HCCDB1, HCCDB3, HCCDB6, HCCDB7, HCCDB12, HCCDB13, HCCDB15, HCCDB16, HCCDB18) and *MUC1* (HCCDB11, HCCDB13). However, we found that the expression of *CD34* was upregulated in HCCDB1, HCCDB3, HCCDB4, HCCDB6, HCCDB7, HCCDB12, HCCDB13, HCCDB15, HCCDB16 and HCCDB18. The expression of *MTOR* was upregulated in HCCDB1, HCCDB3, HCCDB4, HCCDB6, HCCDB7, HCCDB13, HCCDB17 and HCCDB18. The expression of *CSF1* was upregulated in HCCDB4 and HCCDB17.

**Figure 5:**
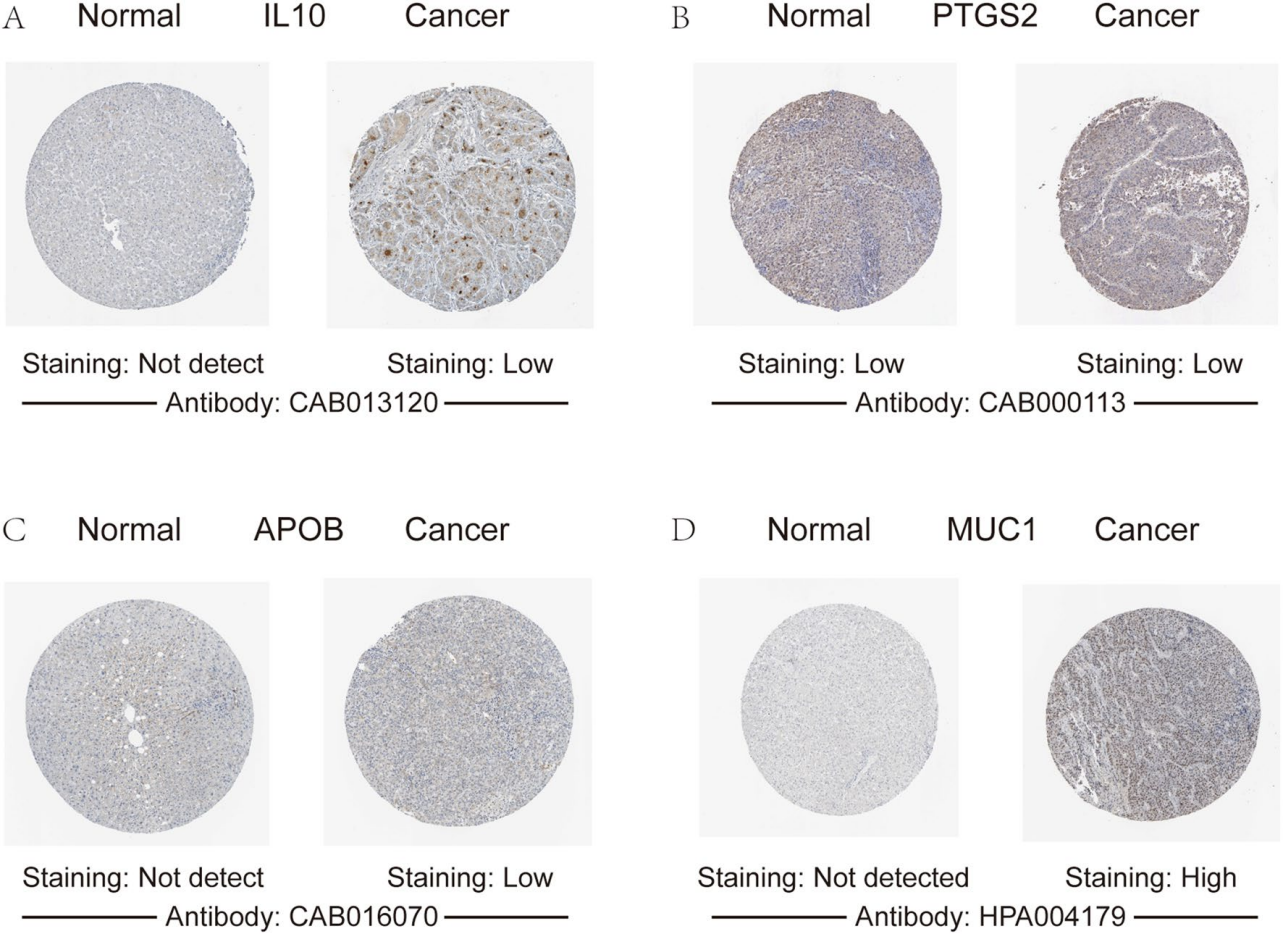
Immunohistochemistry images of four genes in HCC tissues and normal liver tissues. (**A**) IL10, (**B**) PTGS2, (**C**) APOB, (**D**) MUC1.

The immunohistochemical images for four genes (*IL10, PTGS2, APOB and MUC1*) were obtained from the Human Protein Atlas database. These results were consistent with the above results (Fig. 5).

### Correlations between mRNA expression of hub genes and clinical parameters in HCC patients

**Figure 6:**
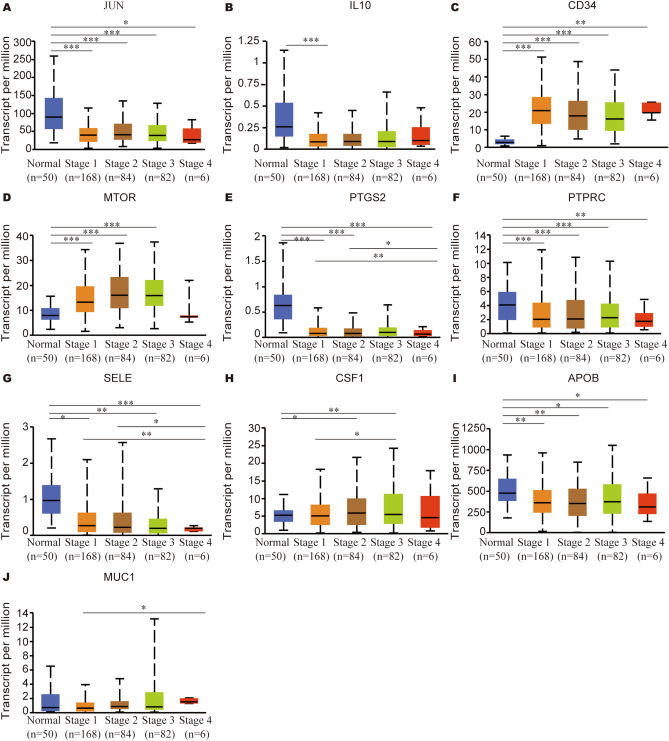
Relationship between mRNA expression of hub genes and individual cancer stages of HCC patients. (**A**) JUN, (**B**) IL10, (**C**) CD34, (**D**) MTOR, (**E**) PTGS2, (**F**) PTPRC, (**G**) SELE, (**H**) CSF1, (**I**) APOB, (**J**) MUC1. *P < 0.05, **P < 0.01, ***P < 0.001.

**Figure 7:**
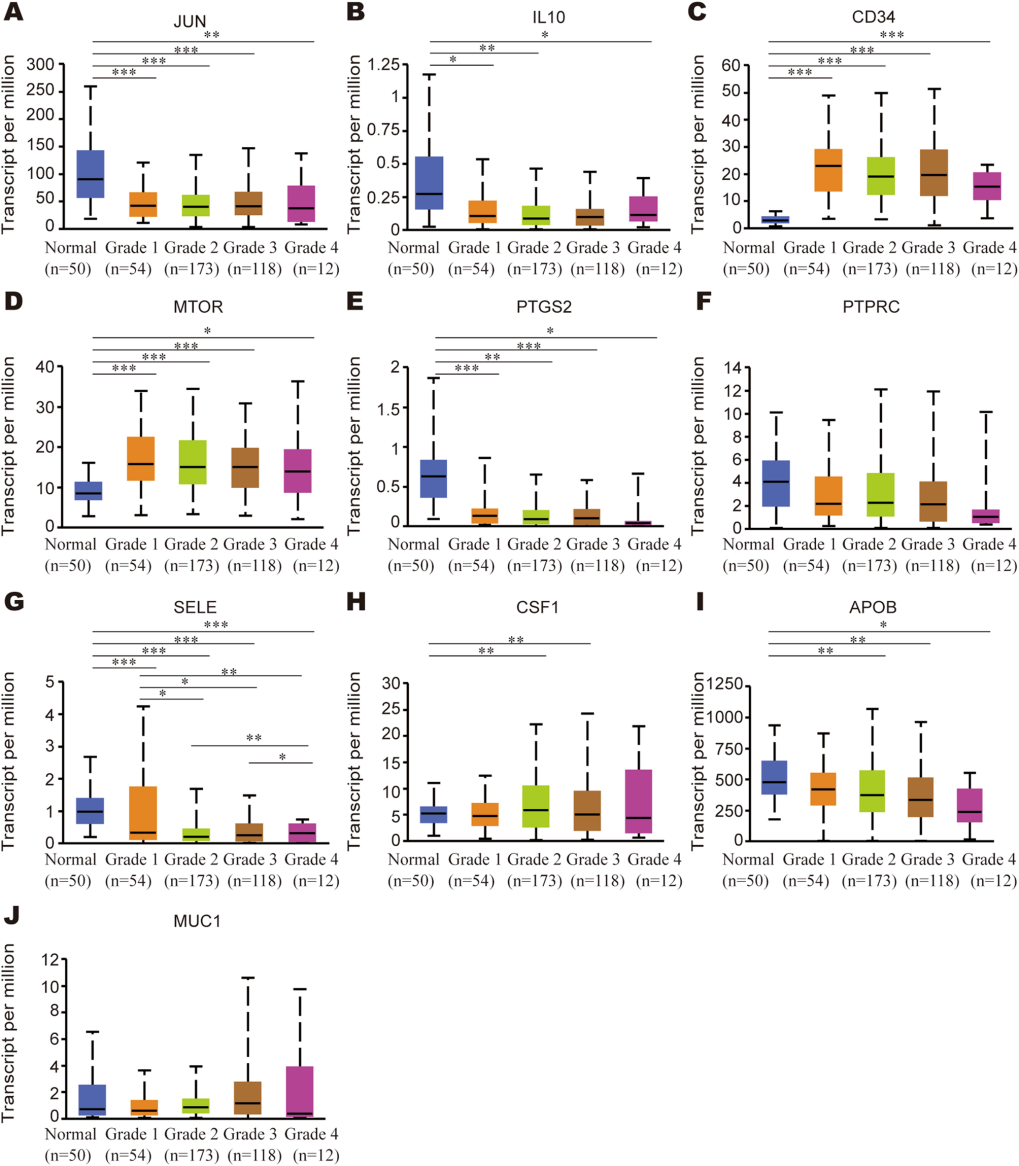
Association of mRNA expression of hub genes with tumor grades of HCC patients. (**A**) JUN, (**B**) IL10, (**C**) CD34, (**D**) MTOR, (**E**) PTGS2, (**F**) PTPRC, (**G**) SELE, (**H**) CSF1, (**I**) APOB, (**J**) MUC1. *P < 0.05, **P < 0.01, ***P < 0.001.

For the relationships between mRNA expression of hub genes and clinical parameters in HCC patients were analyzed by UALCAN, such as patients’ individual cancer stages and tumor grades. As was displayed in Fig. 6, mRNA expression of hub genes was significantly associated with patients’ individual cancer stages. The results indicated that the lowest or highest mRNA expression for the vast majority of hub genes was found in patients with advanced cancer stages. The lowest mRNA expression of *JUN/PTGS2/PTPRC/SELE/APOB* were found in stage 4 (Fig. 6A,E–G,I), while the lowest mRNA expression of *IL10* was found in stage 1 (Fig. 6B). The highest mRNA expression of *CSF1/MUC1* was found in stage 4 (Fig. 6H,J), while the highest mRNA expression of *CD34* and *MTOR* were found in stage 1 and stage 3, respectively (Fig. 6C,D). Similarly, Fig. 7 demonstrated that the mRNA expression of 8 hub genes was remarkably correlated with tumor grades (Fig. 7A–E,G,I). No significant results were observed for PTPRC and MUC1.

**Figure 8:**
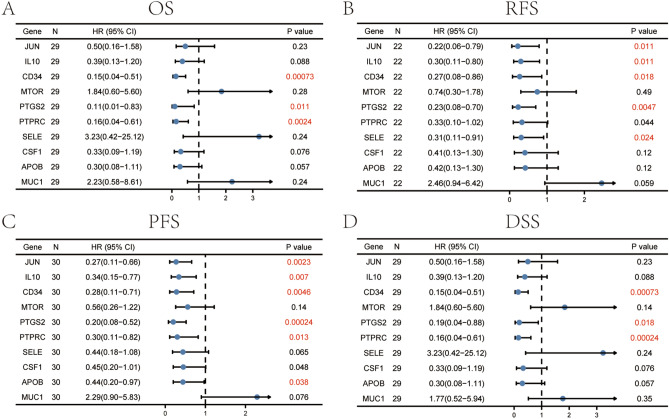
The prognostic value of ten hub genes in HCC patients who received sorafenib treatment. (**A**) OS analyses, (**B**) RFS analyses, (**C**) PFS analyses, (**D**) DSS analyses.

We also explored the prognostic value of hub genes in HCC who received sorafenib treatment (Fig. 10). The results demonstrated that high expression of *CD34/PTGS2/PTPRC* was correlated with favorable OS (Fig. 8A), high expression of *JUN/IL10/CD34/PTGS2/SELE* was correlated with better RFS (Fig. 8B), high expression of *JUN/IL10/CD34/PTGS2/PTPRC/APOB* was correlated with better PFS (Fig. 8C), high expression of *CD34/PTGS2/PTPRC* associated with better DSS (Fig. 8D). Therefore, these hub genes can be used as prognostic indicators for HCC patients who treated by sorafenib.

In addition, higher combinatory mRNA expression of all ten hub genes was associated with gender, race, alcohol consumption, hepatitis virus, stage, grade, AJCC_T and vascular invasion in HCC (Table 2). These results indicated that combinatory mRNA expression of ten hub genes has a better prognosis in the respective clinical parameters for HCC patients.

**Table 2:** Higher combinatory mRNA expressions of all ten hub genes were associated with clinical characteristic in HCC.

Characteristic	N	Low expression of hub genes	High expression of hub genes	Hazard ratio	P value
**Gender**					
Male	246	84	162	0.35 (0.22–0.55)	1.40E − 06
Female	118	74	44	0.66 (0.37–1.20)	0.17
**Race**					
White	181	121	60	0.69 (0.42–1.12)	0.13
Asian	155	83	72	0.17 (0.08–0.38)	1.10E − 06
**Alcohol consumption**					
Yes	115	46	69	0.54 (0.29–1.02)	0.053
No	202	112	90	0.40 (0.24–0.66)	0.00023
**Hepatitis virus**					
Yes	150	79	71	0.29 (0.14–0.62)	0.00068
No	167	113	54	0.54 (0.32–0.89)	0.015
**Stage**					
1 + 2	253	126	127	0.43 (0.26–0.71)	0.00067
3 + 4	87	25	62	0.37 (0.20–0.67)	0.00064
**Grade**					
1	55	34	21	0.40 (0.14–1.16)	0.082
2	174	55	119	0.42 (0.25–0.71)	0.00078
3	118	72	46	0.25 (0.12–0.55)	0.00018
4	12	–	–	–	–
**AJCC_T**					
1	180	62	118	0.42 (0.23–0.75)	0.0024
2	90	35	55	0.51 (0.25–1.05)	0.062
3	78	23	55	0.33 (0.17–0.62)	0.00034
4	13	–	–	–	**–**
**Vascular invasion**					
No	203	68	135	0.47 (0.28–0.79)	0.0035
Micro	90	28	62	0.42 (0.19–0.91)	0.023
Macro	16	–	–	0.33 (0.17–0.62)	–

### Prognostic value of hub genes expression in HCC patients

**Figure 9:**
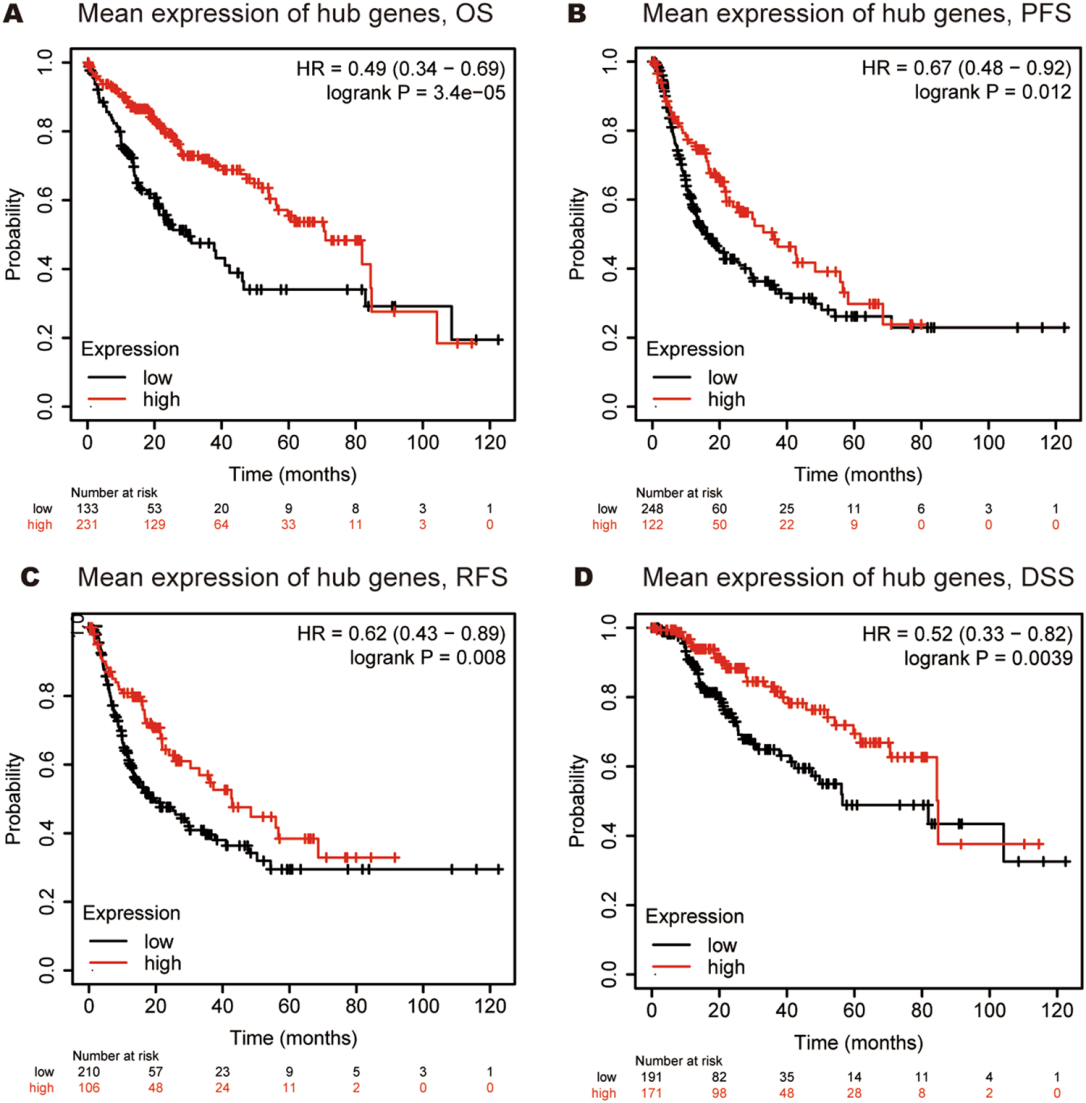
Higher combinatory mRNA expressions of all ten hub genes were associated with better prognosis in HCC. (**A**) OS analysis, (**B**) PFS analysis, (**C**) RFS analysis and (**D**) DSS analysis.

**Figure 10:**
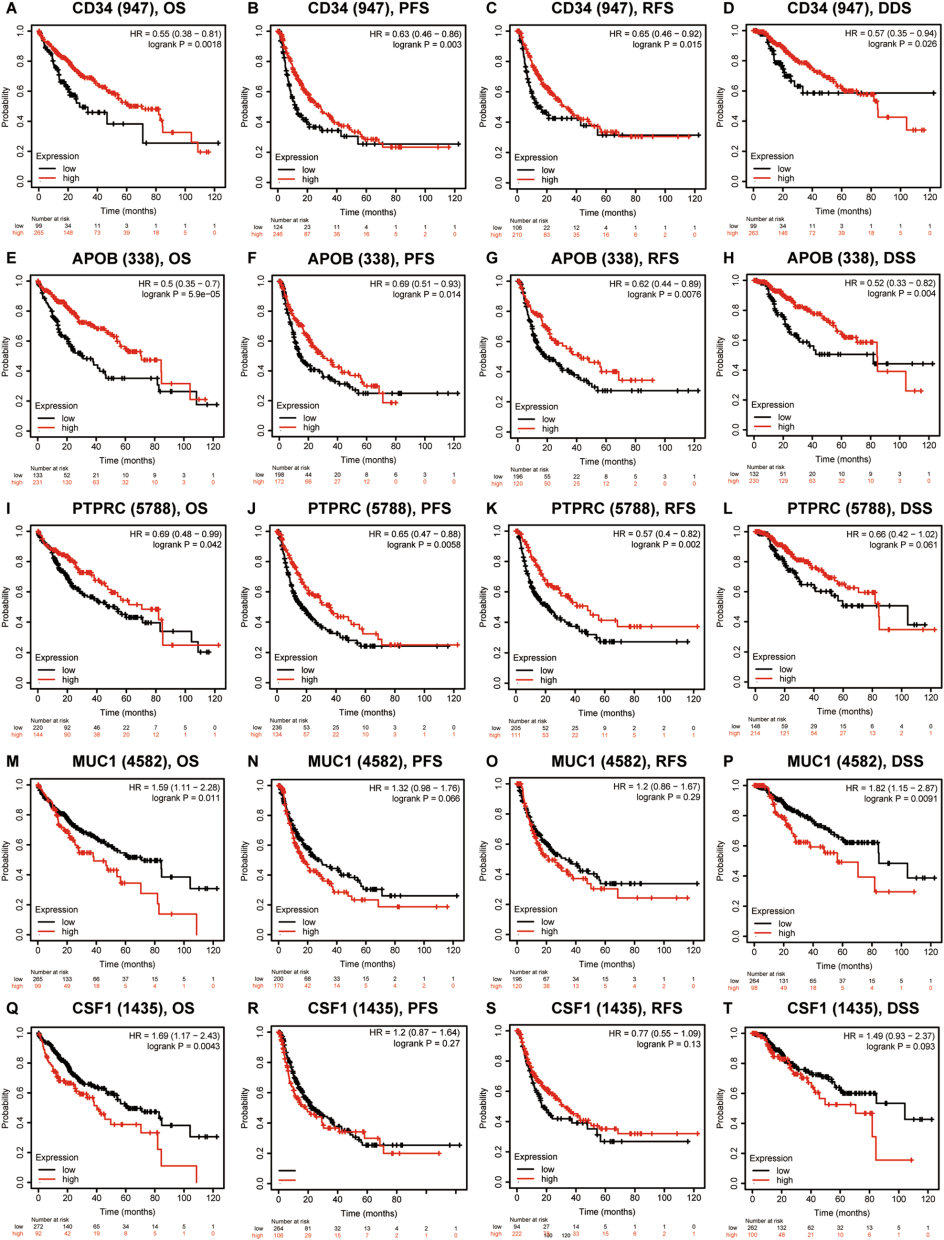
The prognostic value of hub genes in HCC patients. (**A**–**D**) CD34, (**E**–**H**) APOB, (**I**–**L**) PTPRC, (**M**–**P**) MUC1, (**Q**–**T**) CSF1.

We then used Kaplan–Meier plotter to perform survival state of ten hub genes in HCC patients. We analyzed the association between combinatory mRNA expression of ten hub genes and prognosis of HCC patients (Fig. 9). Our results revealed that higher combinatory mRNA expression of ten hub genes was remarkably correlated with favorable OS (HR = 0.49, 95% CI 0.34–0.69, P = 3.4E − 06), PFS (HR = 0.67, 95% CI 0.48–0.92, P = 0.012), RFS (HR = 0.62, 95% CI 0.43–0.89, P = 0.008) and DSS (HR = 0.52, 95% CI 0.33–0.82, P = 0.0039) in HCC patients. Next, we found that high expression of *CD34/APOB/PTPRC* were remarkably correlated with favorable OS of HCC patients (Fig. 10A,E,I), while high expression of *MUC1/CSF1* were remarkably correlated with unfavorable OS of HCC patients (Fig. 10M,Q). Similarly, HCC patients with high expression of *CD34/APOB/PTPRC/SELE/IL10* were remarkably correlated with better PFS (Fig. 10B,F,J; Supplementary Fig. S1B,F) and RFS (Fig. 10C,G,K; Supplementary Fig. S1C,G). High expression of *CD34/APOB/SELE* was associated with better DSS (Fig. 10D,H; Supplementary Fig. S1D), nonetheless, the patients with high expression of *MUC1* were associated with worse DSS (Fig. 10P). No other significant results were observed in Fig. 10L,N,O,R–T.

Additionally, we also performed genetic variations of hub genes in 1026 HCC patients using the cBioportal database. The results indicated that genetic variations of hub genes occurred in 203 (20%) of queried HCC patients. Besides, the percentages of genetic variations in hub genes varied from 0.5 to 10% for individual genes (JUN, 0.5%; IL10, 4%; CD34, 4%; MTOR, 3%; PTGS2, 4%; PTPRC, 6%; SELE, 5%; CSF1, 0.7%; APOB, 10%; MUC1, 6%) (Supplementary Fig. S2A). Genetic alteration of hub genes was shown to be associated with worse OS (P = 0.001098) and DSS (P = 0.0202) for HCC patients (Supplementary Fig. S2B,C).

### Association between hub genes and immune cell infiltration in HCC

For further understand the relationship between hub genes and immune cell infiltrations in HCC, we used TIMER2.0 to explore their relationship. As showed in Table 3, significant correlations between each of the hub genes and tumor purity were found in HCC tissues. Especially, these ten genes showed remarkable correlations with infiltrating levels of B cells, CD8+ T cells, CD4+ T cells, macrophages, neutrophil and DC. These correlation coefficients (COR) were listed from low to high for B cells (0.171–0.375; P < 0.05), CD8+ T cells (0.118–0.396; P < 0.05), CD4+ T cells (0.19–0.278; P < 0.05), macrophages (0.207–0.473; P < 0.05), neutrophil (0.14–0.452; P < 0.05) and DCs (0.138–0.595; P < 0.05). these results demonstrated that hub genes were remarkably associated with tumor-associated B cells, CD8+ T cells, CD4+ T cells, macrophages, neutrophil and DCs in the HCC microenvironment.

**Table 3:** Correlation analysis between candidate hub genes and immune cells in the TIMER2.0 database.

Hub genes	Purity		B cell		CD8^**+**^ T cell		CD4^**+**^ T cell		Macrophage		Neutrophil		DC	
	COR	P	COR	P	COR	P	COR	P	COR	P	COR	P	COR	P
JUN	0.010	0.849	− 0.038	0.48	0.235	< 0.001	− 0.002	0.975	0.281	< 0.001	0.276	< 0.001	0.179	< 0.001
IL10	− 0.472	< 0.001	0.299	< 0.001	0.396	< 0.001	0.085	0.117	0.395	< 0.001	0.293	< 0.001	0.581	< 0.001
CD34	− 0.256	< 0.001	0.164	0.002	0.275	< 0.001	0.190	< 0.001	0.207	< 0.001	0.140	0.009	0.283	< 0.001
MTOR	0.045	0.405	− 0.032	0.560	0.142	0.008	0.000	0.996	0.210	< 0.001	0.225	< 0.001	0.151	0.005
PTGS2	− 0.501	< 0.001	0.166	0.002	0.227	< 0.001	0.255	< 0.001	0.452	< 0.001	0.314	< 0.001	0.419	< 0.001
PTPRC	− 0.444	< 0.001	0.375	< 0.001	0.555	< 0.001	0.205	< 0.001	0.473	< 0.001	0.399	< 0.001	0.595	< 0.001
SELE	− 0.310	< 0.001	− 0.179	< 0.001	0.224	< 0.001	− 0.115	0.033	0.104	0.055	0.141	0.009	− 0.081	0.133
CSF1	− 0.333	< 0.001	0.171	0.001	0.284	< 0.001	0.160	0.003	0.472	< 0.001	0.452	< 0.001	0.509	< 0.001
APOB	0.095	0.079	− 0.176	0.001	0.118	< 0.001	− 0.132	0.028	− 0.007	0.904	0.029	0.588	− 0.138	0.010
MUC1	− 0.216	< 0.001	0.288	< 0.001	0.062	0.252	0.278	< 0.001	0.260	< 0.001	0.297	< 0.001	0.384	< 0.001

CD274, PDCD1 and CTLA4 are vital immune checkpoints that are responsible for tumor immune escape. The associations of hub genes with CD274, PDCD1 and CTLA4 were evaluated. Hub genes expression was markedly associated with CD274 (spearman correlation coefficient ranging from 0.16 to 0.61), CTLA4 (spearman correlation coefficient ranging from 0.13 to 0.69) and PDCD1 (spearman correlation coefficient ranging from 0.16 to 0.62) (Supplementary Fig. S3A–J). These findings demonstrated that these hub genes may play an important role in tumor immune escape in HCC patients.

### mRNA expression of hub genes with high level of infiltrated macrophages predicted unfavorable OS in HCC

**Figure 11:**
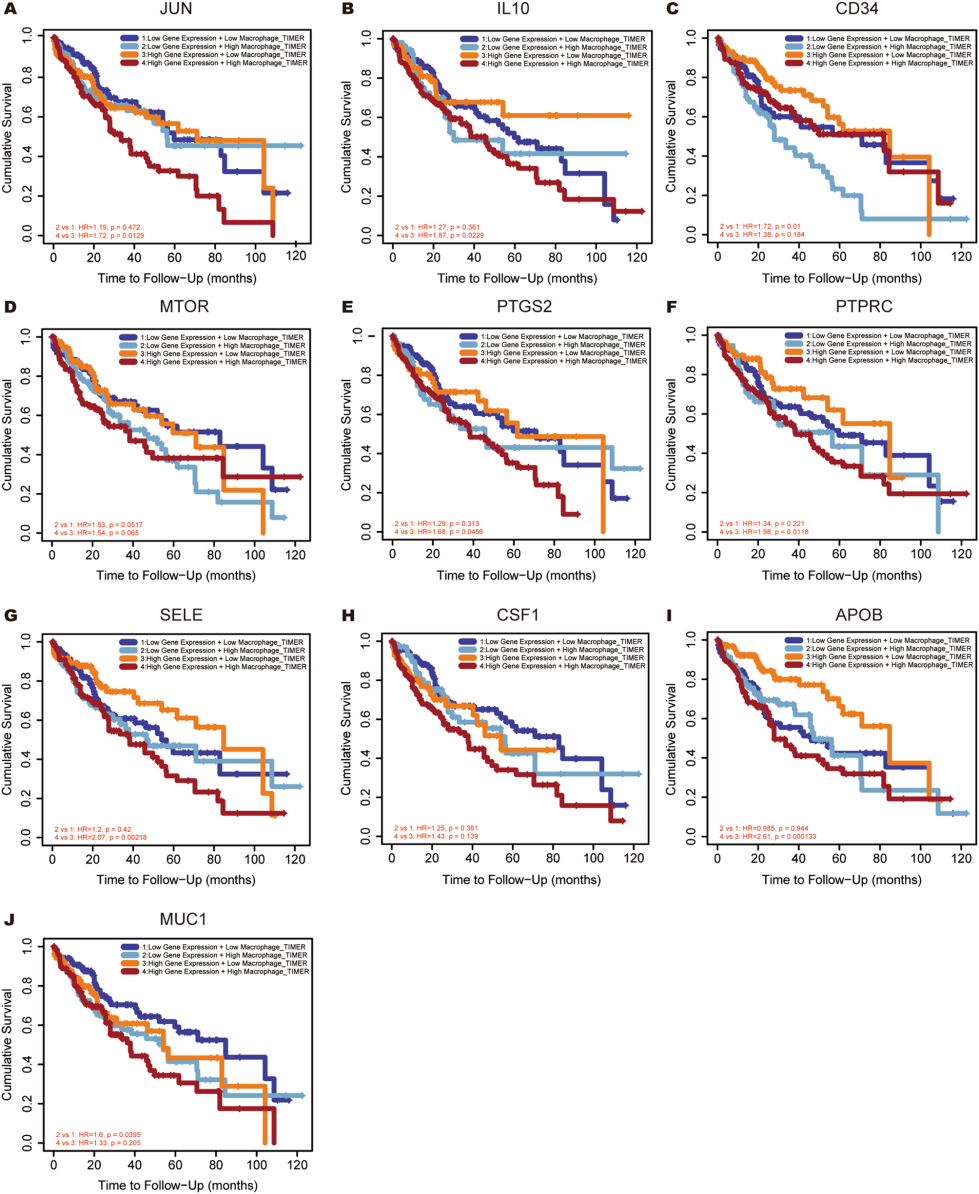
Overall survival analyses for combining the expression of single hub gene with macrophage in HCC patients. (**A**) JUN, (**B**) IL10, (**C**) CD34, (**D**) MTOR, (**E**) PTGS2, (**F**) PTPRC, (**G**) SELE, (**H**) CSF1, (**I**) APOB, (**J**) MUC1.

**Figure 12:**
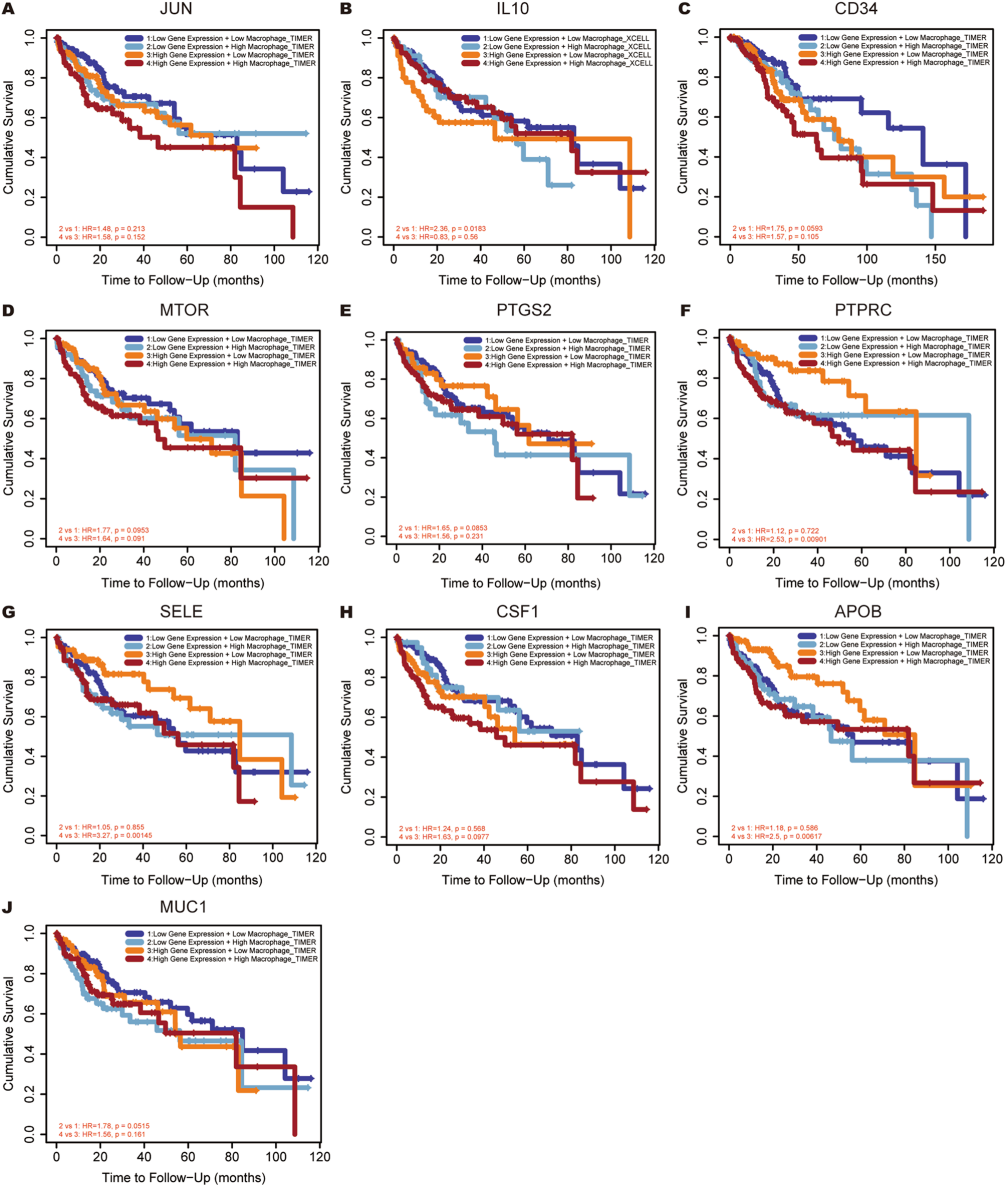
Overall survival analyses for combining the expression of single hub gene with macrophage in HCC patients after adjusting five confounding factors, including age, stage, gender, race, and tumor purity. (**A**) JUN, (**B**) IL10, (**C**) CD34, (**D**) MTOR, (**E**) PTGS2, (**F**) PTPRC, (**G**) SELE, (**H**) CSF1, (**I**) APOB, (**J**) MUC1.

Tumor associated macrophages (TAMs), known as macrophages infiltrating tumors, contribute to tumor initiation, progression, and metastasis. Each of hub genes combined with TAMs was evaluated in prognostic efficiency of HCC patients in this present study. The results were showed in Fig. 11, low expression of *CD34* and *MUC1* with higher macrophage levels intended to show a worse outcome in HCC (HR = 1.72, P = 0.01 for *CD34*; HR = 1.60, P = 0.0395 for *MUC1*). However, under high expression of *JUN/IL10/PTGS2/PTPRC/SELE/APOB*, higher macrophage levels intended to show a worse outcome in HCC (HR = 1.72, P = 0.0129 for *JUN*; HR = 1.87, P = 0.0229 for *IL10*; HR = 1.68, P = 0.0456 for *PTGS2*; HR = 1.98, P = 0.0118 for *PTPRC*; HR = 2.07, P = 0.00218 for *SELE*; HR = 2.61, P = 0.000133 for *APOB*). Moreover, ten hub genes multivariate Cox proportional hazard models were constructed by adjusted for tumor stage, age, race, gender, macrophage level and tumor purity (Fig. 12). The results showed that, under low expression of *IL10*, higher macrophage levels intended to have an unfavorable outcome in HCC (HR = 2.36, P = 0.0183). Furthermore, the high level of TAMs could also predict worse prognosis under the high expression of *PTPRC* (HR = 2.53, P = 0.00901)/*SELE* (HR = 3.27, P = 0.00145)/*APOB* (HR = 2.5, P = 0.00617). These results suggested that seven hub genes were independent prognostic biomarker for HCC patients and four hub genes combined with the TAMs would contribute to the prognosis of HCC.

### Drug selection

We screened 147 drugs for interaction with hub genes through the DGIdb database. Among them, 20 targeted JUN, 12 targeted IL10, 1 targeted CD34, 32 targeted MTOR, 53 targeted PTGS2, 10 targeted PTPRC, 4 targeted SELE, 1 targeted CSF1 and 14 targeted APOB. No candidate drugs with interaction with MUC1 were identified (Supplementary Table S2).

## Discussion

HCC is frequently diagnosed in advanced stages due to its high heterogeneity^34,35^. Currently, surgical resection is the first choice for HCC treatment. However, its effectiveness for HCC treatment is still unsatisfactory^36,37^. Thus, there is an urgent need for identification of novel therapeutic targets and biomarkers in HCC treatment. In our study, multi-databases were comprehensively applied in identifying crucial genes that associated with immune cell infiltrations in HCC. These genes were considered as independent prognostic biomarkers for HCC patients.

A total of 117 common genes were extracted from the intersection of the four databases (GeneCards, DISEASES, CTD and OMIM). The enrichment analysis of BP manifested that these common genes were mainly enriched in positive regulation of cell death, response to toxic substance, response to lipopolysaccharide and positive regulation of cell adhesion. The MF was mainly enriched in transcription coregulatory activity, antioxidant activity, kinase binding and R-SMAD binding. The CC was mainly enriched in membrane raft, perinuclear region of cytoplasm, lysosomal lumen and vesicle lumen. For pathway analysis, the common genes were particularly enriched in pathways in cancer, proteoglycans in cancer, T cell receptor signaling pathway and TNF signaling pathway. These GO terms and KEGG pathway analysis demonstrated that the common genes were enriched in regulating cell function, indicating their close association with tumorigenesis. We then constructed the PPI network based on these genes by STRING, and sub-network of the highest ten degree was identified from the CytoHubba. Next, we explored the mRNA expression of ten hub genes in UALCAN and HCCDB databases, *JUN/IL10/PTGS2/PTPRC/SELE/APOB* were remarkably downregulated in HCC, while *CD34/MTOR/CSF1/MUC1* were upregulated. In addition, we also explored the protein expression of these genes (*IL10, PTGS2, APOB and MUC1*), the results were consistent with mRNA expression results. There were strong relationships between mRNA expression of hub genes and cancer stages and tumor grades from the results of our study, these hub genes can also be used as prognostic indicators for HCC patients who treated by sorafenib. Meanwhile, combinatory mRNA expression of all ten hub genes was associated with clinical parameters in HCC patients, including gender, race, alcohol consumption, hepatitis virus, stage, grade, AJCC_T and vascular invasion. Furthermore, survival curves analyses by Kaplan–Meier plotter showed that higher combinatory mRNA expression of ten hub genes was correlated with favorable OS, PFS, RFS and DSS in HCC patients. However, genetic alteration of hub genes was shown to be associated with worse OS and DSS for HCC patients. We then explored the prognostic value of single hub genes in HCC patients. Our results suggested that *IL10/CD34/PTPRC/SELE/CSF1/APOB/MUC1* had a strong prognostic value for HCC.

These hub genes were confirmed to be strongly associated with infiltrated immune cells in the TIMER2.0 database. These hub genes showed remarkably associated with immune checkpoints (CD274, PDCD1 and CTLA4), which suggested that these hub genes may play an important role in tumor immune escape in HCC patients^38^. Tumor associated macrophages (TAM), known as macrophages infiltrating tumors, contribute to tumor initiation, progression, and metastasis^39,40^. Several studies showed that TAM extremely promotes tumor angiogenesis, resulting in a poor prognosis in HCC^41,42^. The identification of TAM-related genes assists in offering precision therapy for HCC and improving the prognosis of HCC. Thus, we then combined the expression of hub genes with TAM expression to explore the prognostic value in HCC. Under the high expression of *JUN/IL10/PTGS2/PTPRC/SELE/APOB*, high TAM levels predicted unfavorable prognosis. Meanwhile, under the low expression of *CD34/MUC1*, high TAM levels predicted unfavorable prognosis. Furthermore, the multivariate Cox regression models demonstrated that *IL10/PTPRC/SELE/APOB* were independent prognostic biomarker of HCC patients and combined with the TAM would contribute to serving as an important role in clinical prediction of HCC.

*IL10*, an anti-inflammatory and immunosuppressive factor, is a multifunctional cytokine produced by various immune cells^43^. It has been showed to modulate cell growth and differentiation. Previous studies suggested that *IL10* can activate not only immune cells and immune functions, but also limitation of tumor occurrence and progression under specific microenvironments^44,45^. *IL10* was proved to be an independent predictive survival factor for patients diagnosed with HCC. Various studies suggested that high *IL10* expression was correlated with unfavorable prognosis of HCC^46,47^. However, our study provided evidence against such a conclusion. Results from our study showed that high *IL10* expression was correlated with favorable prognosis of HCC. The results were in consistent with the previous studies^48,49^. Furthermore, after adjustments of tumor stage, age, race, gender and tumor purity, under low expression of *IL10*, higher macrophage levels intended to show a worse prognosis of HCC. The potential biological function was that TAM inhibits T cell activation and proliferation through *IL10* to suppress anti-tumor immunity and promote tumor neovascularization^50^.

*PTPRC*, also known as *CD45*, encodes for a protein and belongs to the protein tyrosine phosphatase (PTP) family. Previous study showed that *PTPRC* regulates a variety of cellular processes, such as cell growth, differentiation, oncogenic transformation and mitosis^51^. Recently, several studies have revealed that *PTPRC* is associated with rheumatoid arthritis^52^, systemic lupus erythematosus^53^, Parkinson^54^, multiple sclerosis^55^ and T-cell acute lymphoblastic leukemia^56^. At present, we found that two studies were related to the relationship between *PTPRC* combined with other genes and HCC in animal experiments^57,58^. However, there were few relevant clinical studies. Our study suggested that *PTPRC* combined with the TAM would contribute to acting as an important role in clinical prediction of HCC. Although some possible mechanisms have been proposed, further research is needed^59^.

*SELE*, a member of selectin family, usually expressed on activated platelets and endothelial cells, exerts its effects in lymphocytes and monocyte recruitment, rolling, and diapedesis to the inflammatory areas^60,61^. Previous studies have demonstrated that *SELE* is associated with coronary artery disease^62^, coronary heart disease^63^, coal workers' pneumoconiosis^64^, hypertension^65^ and colorectal cancer^66^. However, there was currently no research on *SELE* gene associated with HCC. Our study indicated that *SELE* gene was as a characteristic prognostic biomarker of HCC. Although the underlying this mechanism remains unknown, the mechanism may involve pathways in cancer related to HCC.

*APOB*, belongs to the apolipoprotein family, forms sub-microscopic spherical particles, which transports dietary lipids from the intestine to the liver via the bloodstream ^67^. Interestingly, multiple studies have indicated that *APOB* is associated with non-small cell lung cancer^68^, gallbladder cancer^69^, low-grade glioma^70^ and primary small cell carcinoma of the esophagus^71^. Study by Lee et al. showed that *APOB* inactivation is associated with poor outcome in HCC patients^72^. This was consistent with our findings that HCC patients with high expression of *APOB* were strongly associated with better overall survival. To date, the underlying mechanism of this relationship remains unknown. However, patients with familial hypobetalipoproteinemia (FHBL) were previously found pathogenic mutations in *APOB*, which is associated with low-density lipoprotein cholesterol and reducing plasma levels of total cholesterol^73^. Individuals with FHBL attributable to *APOB* mutations are intended to hepatic steatosis, liver cirrhosis, and hepatocarcinoma^74^.

Finally, 147 candidate drugs were found for hub genes through the DGIdb database. The identified drugs could provide reference values for clinical practice when they are validated in vitro studies.

Nevertheless, the present study has several shortcomings. First, further experiments are needed to determine the role of these hub genes in HCC. In addition, although the correlation coefficients between these hub genes and immune cell infiltrations were not absolutely high, the results of this study are credible.

In summary, the ten genes were selected from the PPI network. Most of them were independent prognostic biomarker of HCC patients. Moreover, these genes may exert critical function in HCC progression. In addition, we observed that these genes combined with the TAM would contribute to acting as an important role in clinical prediction of HCC. Overall, these findings suggest that these hub genes may be used as novel prognostic biomarkers for HCC therapy.

## Supplementary Information

Supplementary Information.

## Data Availability

All the data we obtained are from publicly available databases, the detail information has been described in “Materials and methods”. Further inquiries are available from the corresponding author.
