# Supplementary material for: Analysis of multiple databases identifies crucial genes correlated with prognosis of hepatocellular carcinoma
Source: Sci Rep. 2022 May 30;12:9002. doi: 10.1038/s41598-022-13159-4 (PMC9151754; doi:10.1038/s41598-022-13159-4)
Supplement: Supplementary file 1 — Supplementary Information. [file 41598_2022_13159_MOESM1_ESM.pdf]

Table S1. Symbols of 117common genes extracted from 4 databases

Gene name	Gene description
ABCD3	ATP binding cassette subfamily D member 3
ADI1	acireductone dioxygenase 1
AGL	amylo-alpha-1, 6-glucosidase, 4-alpha-glucanotransferase
APOB	apolipoprotein B
ARID1A	AT-rich interaction domain 1A
ARID4B	AT-rich interaction domain 4B
ASAP3	ArfGAP with SH3 domain, ankyrin repeat and PH domain 3
ATAD3A	ATPase family AAA domain containing 3A
ATF3	activating transcription factor 3
BCL10	BCL10 immune signaling adaptor
BTG2	BTG anti-proliferation factor 2
CCNL2	cyclin L2
CD34	CD34 molecule
CD46	CD46 molecule
CD55	CD55 molecule (Cromer blood group)
CDC73	cell division cycle 73
CERS2	ceramide synthase 2
CHD1L	chromodomain helicase DNA binding protein 1 like
CLIC4	chloride intracellular channel 4
CMPK1	cytidine/uridine monophosphate kinase 1
CREB3L4	cAMP responsive element binding protein 3 like 4
CSF1	colony stimulating factor 1
CSF3R	colony stimulating factor 3 receptor
CTSK	cathepsin K
DDX1	DEAD-box helicase 1
DIRAS3	DIRAS family GTPase 3
DNAJB4	DnaJ heat shock protein family (Hsp40) member B4
DNMT3A	DNA methyltransferase 3 alpha
DPY30	dpy-30 histone methyltransferase complex regulatory subunit
DRAM2	DNA damage regulated autophagy modulator 2
DTL	denticleless E3 ubiquitin protein ligase homolog
DUSP10	dual specificity phosphatase 10
EIF2AK2	eukaryotic translation initiation factor 2 alpha kinase 2
EIF2D	eukaryotic translation initiation factor 2D
ENAH	ENAH actin regulator
EPHB2	EPH receptor B2
EPHX1	epoxide hydrolase 1
F11R	F11 receptor
F5	coagulation factor V
FASLG	Fas ligand
FOXD3	forkhead box D3
GSTM1	glutathione S-transferase mu 1

HSPG2	heparan sulfate proteoglycan 2
IL10	interleukin 10
ITLN1	intelectin 1
JTB	jumping translocation breakpoint
JUN	Jun proto-oncogene, AP-1 transcription factor subunit
KCTD3	potassium channel tetramerization domain containing 3
KDM1A	lysine demethylase 1A
KDM5B	lysine demethylase 5B
KIF1B	kinesin family member 1B
KISS1	KiSS-1 metastasis suppressor
LAMB3	laminin subunit beta 3
LAMTOR5	late endosomal/lysosomal adaptor, MAPK and MTOR activator 5
LIN28A	lin-28 homolog A
LMNA	lamin A/C
LMO4	LIM domain only 4
MACF1	microtubule actin crosslinking factor 1
MARCKSL1	MARCKS like 1
MTHFR	methylenetetrahydrofolate reductase
MTOR	mechanistic target of rapamycin kinase
MUC1	mucin 1, cell surface associated
MUTYH	mutY DNA glycosylase
NBPF12	NBPF member 12
NET1	neuroepithelial cell transforming 1
NIT1	nitrilase 1
NRAS	NRAS proto-oncogene, GTPase
NTRK1	neurotrophic receptor tyrosine kinase 1
OTUD7B	OTU deubiquitinase 7B
PARK7	Parkinsonism associated deglycase
PARP1	poly(ADP-ribose) polymerase 1
PDZK1	PDZ domain containing 1
PLA2G2A	phospholipase A2 group IIA
PMVK	phosphomevalonate kinase
PRDM2	PR/SET domain 2
PRDX1	peroxiredoxin 1
PSMB4	proteasome 20S subunit beta 4
PTGS2	prostaglandin-endoperoxide synthase 2
PTPRC	protein tyrosine phosphatase receptor type C
PTPRU	protein tyrosine phosphatase receptor type U
PXN	paxillin
RLF	RLF zinc finger
RPS27	ribosomal protein S27
RRM2	ribonucleotide reductase regulatory subunit M2
RSPO1	R-spondin 1
RUNX3	RUNX family transcription factor 3

S100A11	S100 calcium binding protein A11
S100A14	S100 calcium binding protein A14
S100A8	S100 calcium binding protein A8
S100A9	S100 calcium binding protein A9
SDC1	syndecan 1
SDHB	succinate dehydrogenase complex iron sulfur subunit B
SELE	selectin E
SFN	stratifin
SFPQ	splicing factor proline and glutamine rich
SLC2A1	solute carrier family 2 member 1
SMYD3	SET and MYND domain containing 3
SPRTN	SprT-like N-terminal domain
SYTL1	synaptotagmin like 1
TACSTD2	tumor associated calcium signal transducer 2
TNFRSF25	TNF receptor superfamily member 25
TNFRSF9	TNF receptor superfamily member 9
TP53BP2	tumor protein p53 binding protein 2
TP73	tumor protein p73
TPM3	tropomyosin 3
TPO	thyroid peroxidase
TRIM33	tripartite motif containing 33
TRIM62	tripartite motif containing 62
TSPAN1	tetraspanin 1
TXNIP	thioredoxin interacting protein
UBR4	ubiquitin protein ligase E3 component n-recognin 4
UCHL5	ubiquitin C-terminal hydrolase L5
UROD	uroporphyrinogen decarboxylase
VANGL1	VANGL planar cell polarity protein 1
WDR77	WD repeat domain 77
WNT4	Wnt family member 4
YY1AP1	YY1 associated protein 1

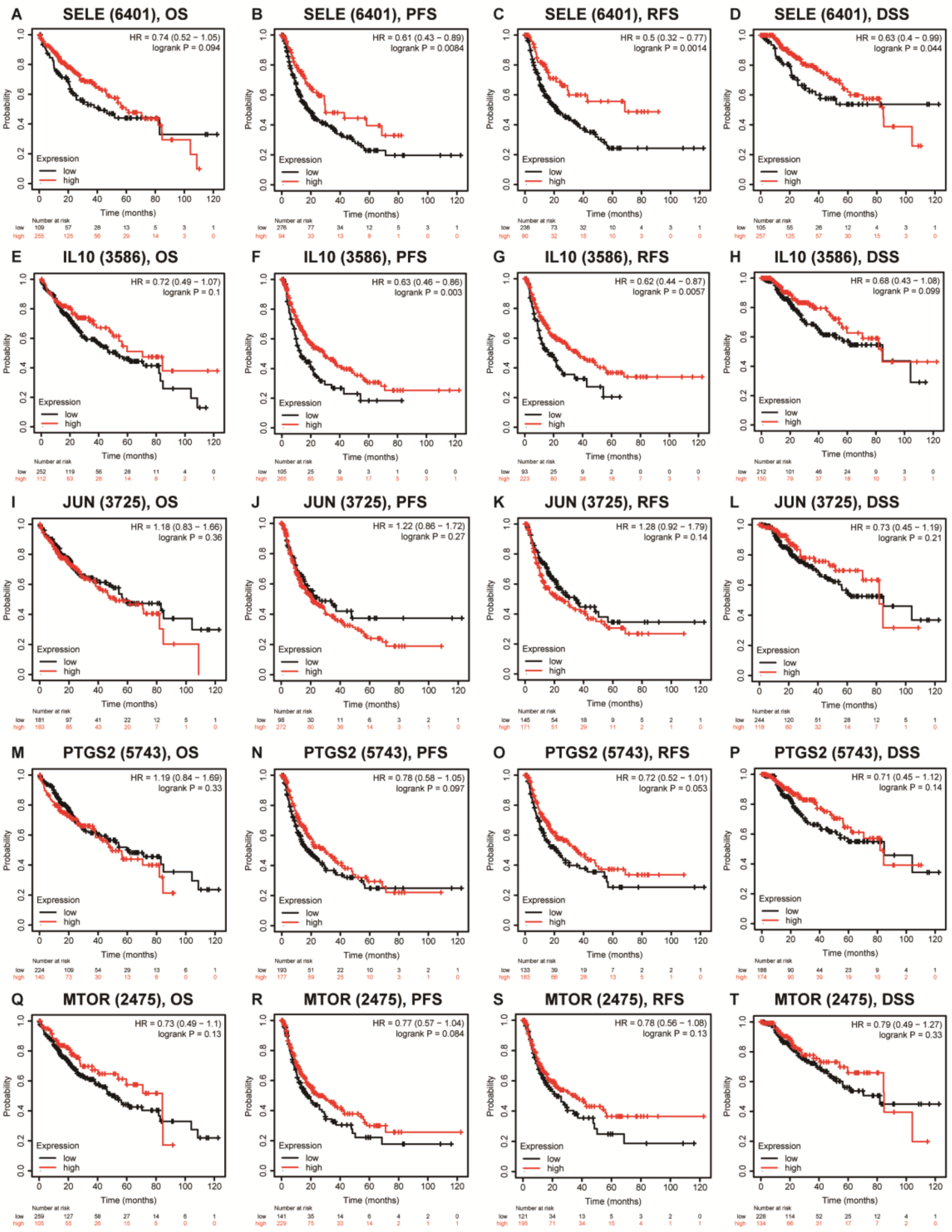

Figure S1 The prognostic value of hub genes in HCC patients. (A-D) SELE, (E-H) IL10, (I-L) JUN, (M-P) PTGS2, (Q-T) MTOR.

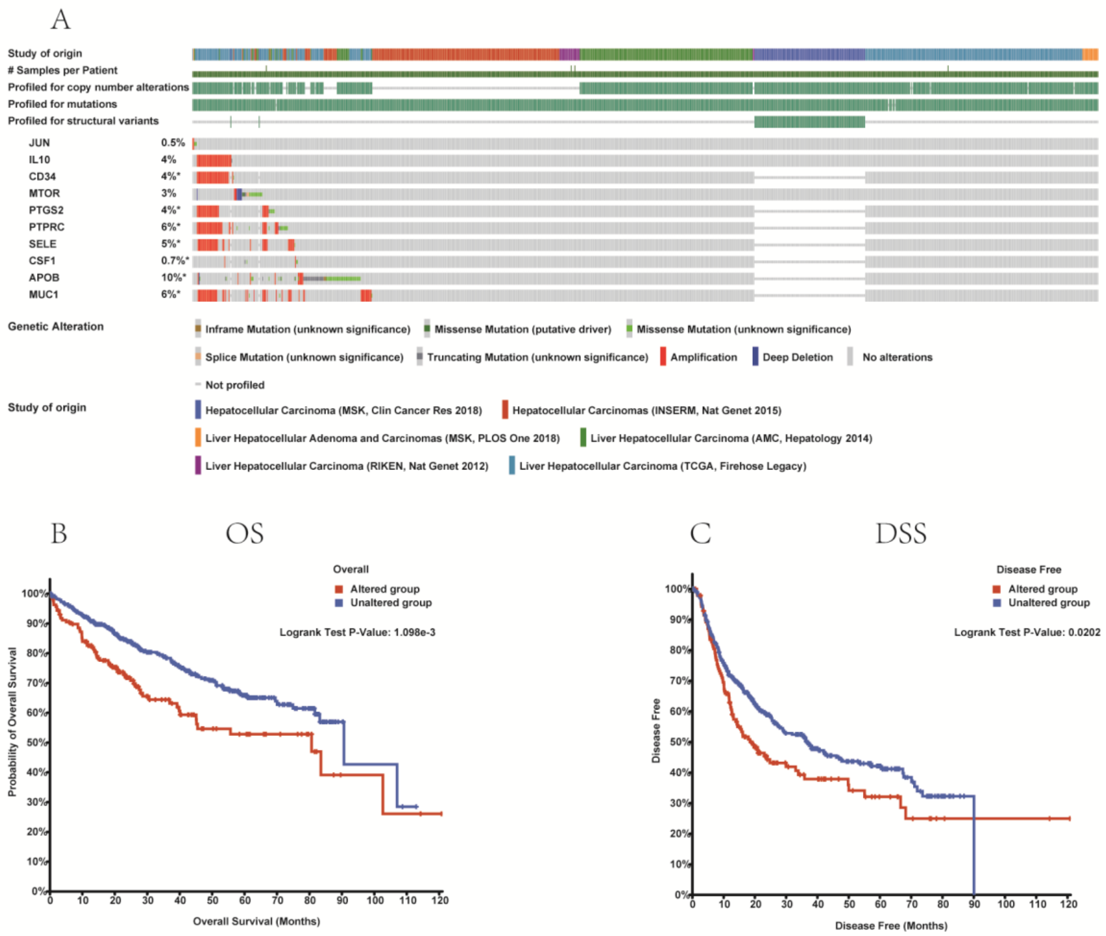

Figure S2. Genetic alterations of hub genes in HCC. (A) Gene alterations of hub genes in HCC. (B-C) Genetic alterations in hub genes were related to shorter OS ($p < 0.01$) and DSS ($p < 0.05$) of HCC patients.

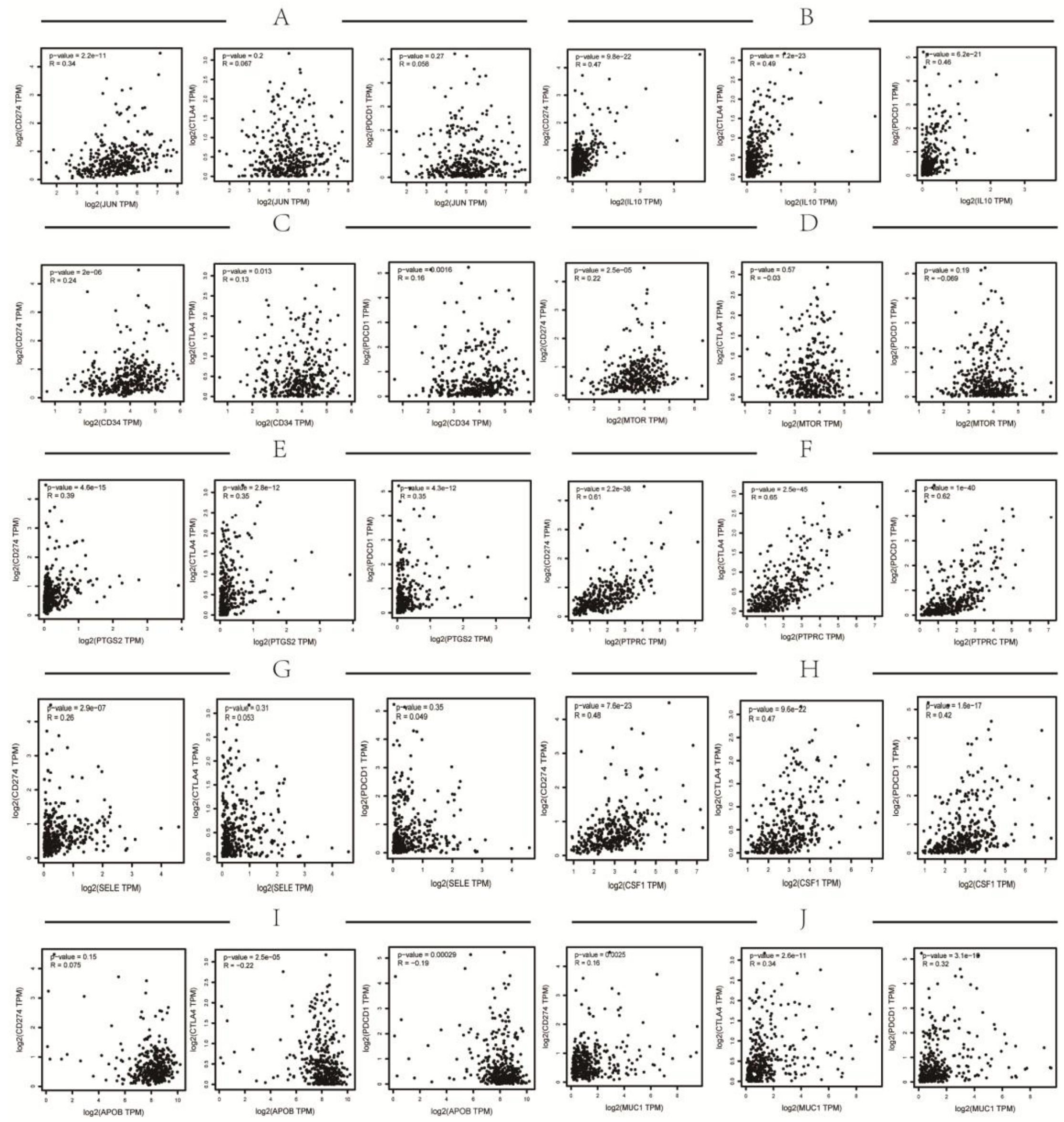

Figure S3. Spearman correlation of hub genes expression with CD274, CTLA4 and PDCD1expression in HCC. (A) JUN, (B) IL10, (C) CD34, (D) MTOR, (E) PTGS2, (F) PTPRC, (G) SELE, (H) CSF1, (I) APOB, (J) MUC1 in HCC.

Table S2. Drugs acting on hub genes

Drug	Gene	Interaction Type & Directionality	Sources	PMIDs	Query Score	Interaction Score
CUPRIC CHLORIDE	JUN	n/a	DTC	None found	4.38	1.45
CIPROFIBRATE	JUN	n/a	DTC	16680159	2.92	0.96
ATOMOXETINE HYDROCHLORIDE	JUN	n/a	DTC	None found	2.19	0.72
BUPROPION HYDROCHLORIDE	JUN	n/a	DTC	None found	1.46	0.48
QUINAPRIL HYDROCHLORIDE	JUN	n/a	DTC	None found	1.46	0.48
CINNARIZINE	JUN	n/a	DTC	16680159	1.25	0.41
AZELASTINE HYDROCHLORIDE	JUN	n/a	DTC	None found	1.09	0.36
DIPHENHYDRAMINE HYDROCHLORIDE	JUN	n/a	DTC	None found	1.09	0.36
MECHLORETHAMINE HYDROCHLORIDE	JUN	n/a	DTC	None found	0.73	0.24
TRIFLUPROMAZINE HYDROCHLORIDE	JUN	n/a	DTC	None found	0.73	0.24
GEMFIBROZIL	JUN	n/a	DTC	16680159	0.73	0.24
FENOFIBRATE	JUN	n/a	DTC	16680159	0.6	0.1
CLOFIBRATE	JUN	n/a	DTC	16680159	0.58	0.19
TROPISETRON	JUN	n/a	DTC	None found	0.44	0.14
SERTRALINE	JUN	n/a	DTC	16680159	0.42	0.14
VINORELBINE TARTRATE	JUN	n/a	DTC	None found	0.27	0.09
CLOTRIMAZOLE	JUN	n/a	DTC	16680159	0.25	0.08
METHIMAZOLE	JUN	n/a	DTC	None found	0.24	0.08

VINBLASTINE SULFATE	JUN	n/a	DTC	None found	0.23	0.08
COLCHICINE	JUN	n/a	DTC	None found	0.09	0.03
ACYCLOVIR	IL10	n/a	NCI	16961277	1.75	1.5
MESALAMINE	IL10	n/a	NCI	15887108	0.88	0.37
RABEPRAZOLE	IL10	n/a	NCI	16815316	0.73	0.62
CLARITHROMYCIN	IL10	n/a	NCI	16815316	0.67	0.58
SIROLIMUS	IL10	n/a	NCI	12907620	0.63	0.27
RITUXIMAB	IL10	n/a	PharmGKB	26384320	0.63	0.53
AMOXICILLIN	IL10	n/a	NCI	16815316	0.51	0.44
EFAVIRENZ	IL10	n/a	PharmGKB	24819158	0.42	0.36
CYCLOSPORINE	IL10	n/a	PharmGKB	18444945	0.28	0.12
ZIDOVUDINE	IL10	n/a	NCI	10433557	0.28	0.24
TRETINOIN	IL10	n/a	NCI	8982293	0.27	0.23
TACROLIMUS	IL10	n/a	PharmGKB	21359536	0.26	0.22
PREDNISOLONE	CD34	n/a	NCI	7532164	0.34	1.22
			TALC			
TEMSIROLIMUS	MTOR	inhibitor (inhibitory)		17935273	3.79	0.42

EVEROLIMUS	MTOR	inhibitor (inhibitory)	TALC	26859683	3.25	0.36
SIROLIMUS	MTOR	inhibitor (inhibitory)	TALC	26619011	3.13	0.17

PIMECROLIMUS	MTOR	potentiator	TdgClinicalTrial	12113647	2.92	0.32
TRIFLUPERIDOL	MTOR	n/a	DTC	None found	2.19	0.24
IFENPRODIL	MTOR	n/a	DTC	None found	2.19	0.24
GLASDEGIB	MTOR	n/a	TTD	None found	2.19	0.24
DOXAZOSIN	MTOR	n/a	DTC	None found	0.88	0.1
PENTAMIDINE ISETHIONATE	MTOR	n/a	DTC	None found	0.73	0.08
LOPERAMIDE	MTOR	n/a	DTC	None found	0.44	0.05
PROPAFENONE	MTOR	n/a	DTC	None found	0.4	0.04
MAPROTILINE	MTOR	n/a	DTC	None found	0.36	0.04
DEQUALINIUM	MTOR	n/a	DTC	None found	0.36	0.04
AMSACRINE	MTOR	n/a	DTC	None found	0.29	0.03
NICARDIPINE	MTOR	n/a	DTC	None found	0.29	0.03
COPANLISIB	MTOR	inhibitor (inhibitory)	GuideToPharmacology	None found	0.24	0.03
MIFEPRISTONE	MTOR	n/a	DTC	None found	0.22	0.02
PERPHENAZINE	MTOR	n/a	DTC	None found	0.22	0.02
PAZOPANIB	MTOR	n/a	CIViC	24625776	0.21	0.02
AMIODARONE	MTOR	n/a	DTC	None found	0.2	0.02
PAROXETINE	MTOR	n/a	DTC	None found	0.18	0.02
NICLOSAMIDE	MTOR	n/a	DTC	None found	0.16	0.02

METFORMIN HYDROCHLORIDE	MTOR	n/a	DTC	23490148	0.16	0.02
EPINEPHRINE	MTOR	n/a	DTC	None found	0.15	0.02
ISOPROTERENOL	MTOR	n/a	DTC	None found	0.15	0.02
PIMOZIDE	MTOR	n/a	DTC	None found	0.15	0.02
ALPELISIB	MTOR	inhibitor (inhibitory)	CancerCommons	None found	0.13	0.01
IDARUBICIN	MTOR	n/a	DTC	None found	0.12	0.01
METFORMIN	MTOR	inhibitor (inhibitory)	MyCancerGenomeClinicalTrial	None found	0.12	0.01
DISULFIRAM	MTOR	n/a	DTC	None found	0.11	0.01
VANDETANIB	MTOR	n/a	DTC	None found	0.08	0.01
HALOPERIDOL	MTOR	n/a	DTC	None found	0.06	0.01
ETORICOXIB	PTGS2	inhibitor (inhibitory)	TdgClinicalTrial	17573128	56.9	9.4
CARPROFEN	PTGS2	inhibitor (inhibitory)	TdgClinicalTrial	15939622	48.15	7.96

ETODOLAC	PTGS2	inhibitor (inhibitory)	TdgClinicalTrial	12824918	18.97	3.13
OXAPROZIN	PTGS2	inhibitor (inhibitory)	TdgClinicalTrial	19338579	14.59	2.41
KETOROLAC	PTGS2	inhibitor (inhibitory)	TdgClinicalTrial	11695255	13.13	2.17
SALSALATE	PTGS2	inhibitor (inhibitory)	TdgClinicalTrial	9711054	13.13	2.17
TOLMETIN	PTGS2	inhibitor (inhibitory)	TdgClinicalTrial	14613550	13.13	2.17
MELOXICAM	PTGS2	inhibitor (inhibitory)	TdgClinicalTrial	10567199	13.13	2.17

KETOPROFEN	PTGS2	inhibitor (inhibitory)	TdgClinicalTrial	14513718	9.63	1.59
FLURBIPROFEN	PTGS2	inhibitor (inhibitory)	DTC	10091674	9.48	1.57
NEPAFENAC	PTGS2	inhibitor (inhibitory)	TdgClinicalTrial	10850857	8.75	1.45
DIFLUNISAL	PTGS2	inhibitor (inhibitory)	TdgClinicalTrial	11315375	8.75	1.45
TENOXICAM	PTGS2	inhibitor (inhibitory)	TdgClinicalTrial	11563332	7.88	1.3

BALSALAZIDE	PTGS2	inhibitor (inhibitory)	TdgClinicalTrial	17981262	6.57	1.08
DEXIBUPROFEN	PTGS2	inhibitor (inhibitory)	DTC	20143779	6.57	1.08
NABUMETONE	PTGS2	inhibitor (inhibitory)	TdgClinicalTrial	11304699	6.25	1.03
NAPROXEN	PTGS2	inhibitor (inhibitory)	TdgClinicalTrial	17604186	5.11	0.84
PARECOXIB	PTGS2	inhibitor (inhibitory)	TdgClinicalTrial	10794682	4.38	0.72
FENOPROFEN	PTGS2	inhibitor (inhibitory)	TdgClinicalTrial	17050798	4.38	0.72
PIROXICAM	PTGS2	inhibitor (inhibitory)	TdgClinicalTrial	11952155	3.58	0.59

MESALAMINE	PTGS2	inhibitor (inhibitory)	TdgClinicalTrial	16855178	3.5	0.29
MEFENAMIC ACID	PTGS2	inhibitor (inhibitory)	ChemblInteractions	10393680	3.06	0.51
FENOPROFEN CALCIUM	PTGS2	inhibitor (inhibitory)	ChemblInteractions	None found	2.19	0.36
AMINOSALICYLATE POTASSIUM	PTGS2	inhibitor (inhibitory)	ChemblInteractions	None found	2.19	0.36
IBUPROFEN LYSINE	PTGS2	inhibitor (inhibitory)	ChemblInteractions	None found	2.19	0.36
DICLOFENAC POTASSIUM	PTGS2	inhibitor (inhibitory)	ChemblInteractions	None found	2.19	0.36
DICLOFENAC EPOLAMINE	PTGS2	inhibitor (inhibitory)	ChemblInteractions	None found	2.19	0.36
OXAPROZIN POTASSIUM	PTGS2	inhibitor (inhibitory)	ChemblInteractions	None found	2.19	0.36
FENBUFEN	PTGS2	n/a	TTD	None found	2.19	0.36
SULINDAC	PTGS2	inhibitor (inhibitory)	TdgClinicalTrial	11118042	2.19	0.36

AMINOSALICYLATE SODIUM	PTGS2	inhibitor (inhibitory)	ChemblInteractions	None found	2.19	0.36
DICLOFENAC	PTGS2	inhibitor (inhibitory)	TdgClinicalTrial	12705061	2.01	0.33
IBUPROFEN	PTGS2	inhibitor (inhibitory)	TdgClinicalTrial	25502615	1.93	0.32
INDOMETHACIN	PTGS2	inhibitor (inhibitory)	DTC	15668944	1.84	0.3
KETOROLAC TROMETHAMINE	PTGS2	inhibitor (inhibitory)	ChemblInteractions	None found	1.46	0.24
NAPROXEN SODIUM	PTGS2	inhibitor (inhibitory)	ChemblInteractions	None found	1.46	0.24

TOLMETIN SODIUM	PTGS2	inhibitor (inhibitory)	ChemblInteractions	None found	1.46	0.24
NIMESULIDE	PTGS2	inhibitor (inhibitory)	GuideToPharmacology	16846549	1.35	0.22
SULFASALAZINE	PTGS2	inhibitor (inhibitory)	TdgClinicalTrial	16855178	1.28	0.21
BALSALAZIDE DISODIUM	PTGS2	inhibitor (inhibitory)	ChemblInteractions	None found	1.09	0.18
BISMUTH SUBSALICYLATE	PTGS2	inhibitor (inhibitory)	ChemblInteractions	None found	1.09	0.18
OLSALAZINE SODIUM	PTGS2	inhibitor (inhibitory)	ChemblInteractions	None found	1.09	0.18
ACETAMINOPHEN	PTGS2	inhibitor (inhibitory)	TdgClinicalTrial	17884974	0.9	0.15
THALIDOMIDE	PTGS2	antagonist (inhibitory)	TdgClinicalTrial	12710892	0.88	0.14
HYDROXYCHLOROQUINE	PTGS2	n/a	NCI	14963695	0.8	0.13
DICLOFENAC SODIUM	PTGS2	inhibitor (inhibitory)	ChemblInteractions	None found	0.73	0.12

BENZQUINAMIDE	PTGS2	inhibitor (inhibitory)	GuideToPharmacology	None found	0.49	0.08
CAPECITABINE	PTGS2	n/a	PharmGKB	19219602	0.49	0.04
OXALIPLATIN	PTGS2	n/a	PharmGKB	19219602	0.47	0.04
ASPIRIN	PTGS2	inhibitor (inhibitory)	ChemblInteractions	17522398	0.36	0.06
RALOXIFENE	PTGS2	n/a	NCI	15243281	0.29	0.05
CYCLOSPORINE	PTGS2	n/a	NCI	10999939	0.28	0.02
ATENOLOL	PTGS2	n/a	PharmGKB	None found	0.13	0.02
HYDROGEN PEROXIDE	PTPRC	n/a	DTC	15012988	8.75	10.61
ALENDRONIC ACID	PTPRC	n/a	DTC	15012988	1.09	1.33
ADALIMUMAB	PTPRC	n/a	PharmGKB	20309874	0.77	0.94
ETANERCEPT	PTPRC	n/a	PharmGKB	20309874	0.69	0.84
INFLIXIMAB	PTPRC	n/a	PharmGKB	20309874	0.66	0.8
EPOETIN BETA	PTPRC	n/a	NCI	1713254	0.63	0.76
HYDROCORTISONE	PTPRC	n/a	NCI	10731548	0.46	0.28
ESTRADIOL	PTPRC	n/a	NCI	10325261	0.29	0.35
PREDNISONE	PTPRC	n/a	NCI	17063711	0.27	0.33
FLUOROURACIL	PTPRC	n/a	NCI	15206578	0.17	0.1
CAPECITABINE	SELE	n/a	PharmGKB	24980946	0.49	0.44
OXALIPLATIN	SELE	n/a	PharmGKB	24980946	0.47	0.43
LEUCOVORIN	SELE	n/a	PharmGKB	24980946	0.31	0.57

FLUOROURACIL	SELE	n/a	PharmGKB	24980946	0.17	0.15
PEXIDARTINIB	CSF1	n/a	CIViC	26222558	0.8	5.79
CHOLESTYRAMINE	APOB	n/a	NCI	3906004	2.92	2.36
MIPOMERSEN	APOB	n/a	TdgClinicalTrial	None found	2.19	1.77
LOMITAPIDE	APOB	n/a	PharmGKB	None found	1.09	0.88
FENOFIBRATE	APOB	n/a	NCI	12606523	0.91	0.37
IRBESARTAN	APOB	n/a	PharmGKB	15614026	0.8	0.64
ATORVASTATIN	APOB	n/a	NCI	11882317	0.73	0.59
HYDROCORTISONE	APOB	n/a	NCI	1597147	0.46	0.19
LOVASTATIN	APOB	n/a	NCI	2351867	0.41	0.33
NEVIRAPINE	APOB	n/a	NCI	12869587	0.38	0.31
TRIFLUOPERAZINE	APOB	n/a	NCI	7595077	0.31	0.25
PRAVASTATIN	APOB	n/a	NCI	8312689	0.25	0.2
WARFARIN	APOB	n/a	PharmGKB	None found	0.12	0.1
ALCOHOL	APOB	n/a	NCI	8576634	0.1	0.08
DEXAMETHASONE	APOB	n/a	NCI	7670964	0.1	0.08
